# Cognitive and motor abilities predict auditory-cued finger tapping in a dual task

**DOI:** 10.3389/fnins.2025.1553548

**Published:** 2025-05-21

**Authors:** Mohammed A. Mudarris, Renske N. Krijt, Allyah M. Hassell, Tanya M. Murphy, Marit F. L. Ruitenberg, Marjolein Fokkema, Rebecca S. Schaefer

**Affiliations:** ^1^Health, Medical, and Neuropsychology Unit, Institute of Psychology, Faculty of Social and Behavioural Sciences, Leiden University, Leiden, Netherlands; ^2^Leiden Institute for Brain and Cognition, Leiden University, Leiden, Netherlands; ^3^Department of Psychology, College of Social Sciences and Media, University of Jeddah, Jeddah, Saudi Arabia; ^4^Department of Psychology, Faculty of Biomedical and Health Sciences, Universidad Europea de Madrid, Madrid, Spain; ^5^Methodology and Statistics Unit, Institute of Psychology, Faculty of Social and Behavioural Sciences, Leiden University, Leiden, Netherlands; ^6^Academy of Creative and Performing Arts, Faculty of Humanities, Leiden University, Leiden, Netherlands

**Keywords:** sensorimotor synchronization, motor timing, tapping force, rhythm, rhythmic movement, cognitive inhibition, music, cognitive-motor interference

## Abstract

Rhythmic auditory stimulation, a therapeutic method involving repetitive movements cued by rhythmic sounds, can support movement (re-)learning and attentional orienting, but effects vary. While properties of cues have been extensively studied, not much is known about the impact of individual differences in cognitive and motor abilities that enable sensorimotor synchronization. This study examined how stimulus complexity (metronome/music) and cognitive and motor functions affect tapping timing consistency and force. Fifty healthy young adults (ages 18–39) performed several finger tapping tasks, specifically, as a stand-alone task (single task), and simultaneously with 2-Back working memory task (dual task), each to sounds with a clear, steady beat and differing levels of rhythmic complexity (metronome vs. music). Standardized neuropsychological tests were related to consistency and force in the single task and to their dual task cost (interference). The dual task led to lower tapping consistency for both auditory cues. Poorer cognitive inhibition and better gross motor ability each predicted greater applied force. In contrast, participants with poorer fine motor ability tapped with lower force. Accounting for musical training and reward from music revealed that sustained attention, verbal memory, musical training and reward additionally predicted tapping force, whereas only musical training predicted better tapping consistency. These non-linear relationships were shown for both single and dual tasks, but not for the dual task cost. Overall, this study highlights the complex interactions of movement functions and cognitive abilities with sensorimotor synchronization, essential to many music-based interventions, and underlines the importance of the consideration of individual baseline abilities.

## 1 Introduction

Sensorimotor synchronization (SMS) refers to the rhythmic movement to periodic sound or cues (Repp and Su, 2013). In healthy individuals, SMS can facilitate attention (Schmidt-Kassow et al., 2013). It also has various clinical applications in both movement and cognitive rehabilitation, where it is typically referred to as rhythmic auditory stimulation (for reviews, see Schaefer, 2014; Schmid, 2024; Thaut, 2013). Examples of these applications are improved attention, memory, and executive functioning in stroke patients (Park and Lee, 2018), as well as decreases in attentional load, and improvement of either cognitive or motor performance in various other neurological populations (Schmid, 2024). Despite common applications of rhythmic auditory stimulation, its effectiveness varies among individuals (e.g., Bella et al., 2018), and the aspects crucial for its success remain unknown. Elements of SMS found to facilitate or hamper someone’s ability to move to sound include the sound that is used, presumably affecting the interactions of cognitive and motor functions needed to synchronize (Wittwer et al., 2013).

Auditory cues for SMS can range from simple metronomes to complex musical pieces (Schaefer, 2014). Metronomes offer a stable, predictable pulse, whereas musical cues generally add rhythmic complexity (Levitin et al., 2018). While music can improve entrainment in timing accuracy of motor responses (Rose et al., 2019), it has also been proposed that more complex stimuli can be unnecessarily distracting (Rose et al., 2021). Metronomes tend to improve movement consistency, measurable as a lower variance of motor intervals, known as the coefficient of variation (CV; Semjen et al., 2000). Comparing music to metronome cued tapping suggests that synchronization improves with lower rhythmic complexity (Tranchant et al., 2016). Moreover, higher ease of perception of the beat or pulse (i.e., beat clarity), appears to improve SMS the most (Miguel et al., 2020; Tranchant et al., 2016).

SMS inherently involves perceptual, motor, and cognitive aspects (Schaefer, 2014). In terms of cognitive aspects, better memory and attention abilities are thought to support synchronization abilities (c.f., Grahn and Schuit, 2012; Guérin et al., 2021). Dual task paradigms can elucidate cognitive aspects of SMS; task performance generally declines for simultaneous cognitive and motor tasks as compared to single tasks. For instance, when performing hand movements while speaking (Pohl et al., 2011), the deterioration in the dual task (DT) relative to the single task (ST) indicates the dual task cost (DTC). This deterioration, conceptually termed cognitive-motor interference (CMI), presumably occurs due to competition for underlying cognitive or neural resources in either domain (Leone et al., 2017). The extent of DTC in SMS is less clear, as both negative effects and facilitation of secondary tasks have been reported. For example, performing a working memory task during SMS impairs motor consistency (Maes et al., 2015), cognitive performance (Guérin et al., 2021), or both (Bååth et al., 2016). However, cognitive-motor entrainment may also improve cognitive or motor performance, hypothesized as decreasing attentional load of movement tasks (Schmid, 2024). CMI can therefore inform our understanding of SMS mechanisms and varying clinical potential. Moreover, while for other forms of movement, higher baseline motor and cognitive capabilities may ameliorate CMI (Yogev et al., 2008), for SMS in DT contexts this is unclear.

SMS research has most often focused on finger tapping, and specifically on cue properties, perceptual aspects, and musical abilities influencing performance (see Levitin et al., 2018), but it remains unclear what individual differences in cognitive and motor abilities contribute to SMS in ST contexts. Interindividual differences in SMS tapping were previously linked to cognitive (Mudarris and Schaefer, 2022; Rabinowitz and Lavner, 2014), and motor abilities (Monier and Droit-Volet, 2019). For instance, better timing in music-cued SMS is predicted by better working memory and inhibitory control (Colley et al., 2018; Grahn and Schuit, 2012; Guérin et al., 2021; Slater et al., 2018; Zalta et al., 2020). These relationships persist even when accounting for fine motor ability (Löser et al., 2024).

While cognitive predictors of SMS during finger tapping have been relatively widely studied, studies assessing the extent to which motor abilities predict tapping performance remain limited. One study observed that fine motor abilities were associated with differences in SMS variability among children (Monier and Droit-Volet, 2019), but another study on adults did not find such an association (Lorås et al., 2013). However, as finger tapping performance and baseline fine motor abilities change across the lifespan (Aoki and Fukuoka, 2010; Drewing et al., 2006; Ruitenberg et al., 2019), and age is linked to declining SMS (Carment et al., 2018), further investigation of the effects of motor abilities on SMS is warranted. More indirect evidence suggesting a link between individual differences in motor abilities and SMS comes from observations that SMS is reduced in people with neurocognitive disorders that affect motor functioning (Navarro-López et al., 2024; von Schnehen et al., 2022). These associations between age-related and clinical deficits in motor function with synchronization call for further examination.

The current study examines how cognitive and motor abilities influence SMS performance in terms of tapping timing consistency and force for different auditory cues (i.e., metronome and music), and cognitive load (ST vs. DT). Specifically, we compare auditory conditions to clarify the effects of rhythmic complexity on timing consistency and tapping force. In a dual-task paradigm, we also examine CMI’s impact on timing consistency and tapping force, for both cue types. Furthermore, we examine whether cognitive abilities (i.e., switching ability, sustained attention, verbal memory and inhibitory control), and motor abilities (i.e., fine and gross) predict SMS performance alone or DTC in combination with a DT, aiming to elucidate the interplay between cognitive and motor abilities in SMS. Preregistered hypotheses^1^ (AsPredicted.org/cw2s-36ns.pdf) state that (1) tapping consistency and tapping force decrease for the DT as compared to the ST condition. Additionally, we hypothesized that (2) better fine and gross motor abilities will predict better tapping consistency. Moreover, (3) switching and inhibitory control will inversely predict tapping force, while attention and verbal memory will positively predict tapping consistency. In addition to examining these associations in the ST, they will be investigated for DTC to assess the role of CMI. Furthermore, we hypothesize that (4) better cognitive and motor abilities are expected to predict lower DTC for both tapping consistency and force. Finally, we hypothesized that (5) reward from music and musical training will positively predict better tapping consistency and higher tapping force.

## 2 Materials and methods

### 2.1 Participants

Fifty healthy young adults (M_*age*_ = 22.22 ± 4.04; 18–39 years; 76% female; see Table 1) were recruited through university networks and social media. Five participants missed tapping data for both conditions due to technical problems or too few taps for analysis. Another participant missed data for only the metronome condition and was retained in the analysis. The final sample for tapping analyses comprised 45 participants (M_*age*_ = 22.49 ± 4.21, range 18–39; 77% females). Sample size was calculated *a priori* based on a similar within-subject study design with a medium effect size (η^2^ = 0.08; Langhanns and Mueller, 2020), indicating that 42 participants were needed for sufficient power [α = 0.05 and (1-β) = 0.95], using G*Power 3.1. Participants completed the study in English or Dutch, and handedness was determined using the Edinburgh Handedness Inventory (Oldfield, 1971). Exclusion criteria were neurological or psychiatric conditions, uncorrected vision or hearing impairments, motor impairments, and color-blindness. The study did not account for educational level and socioeconomic status. The study was approved by The Psychology Research Ethics Committee of the Institute of Psychology at Leiden University (2022-01-05-R.S.Schaefer-V2-3558) and informed consent was obtained in accordance with the Declaration of Helsinki.

**TABLE 1:** Frequency and distribution of participants’ demographic data.

Variable		*n (%)*	*M*	*SD*	*Min*	*Max*
Age		50 (100%)	22.20	4.05	18	39
Gender	Male	12 (24%)				
	Female	38 (76%)				
Education	Education-level – attained		Education-level – aspired			
	Primary	3 (6%)	—			
	Secondary	20 (40%)	—			
	Undergraduate/professional	10 (20%)	23 (46%)			
	Postgraduate	2 (4%)	23 (46%)			
	Ongoing education	15 (30%)	—			
Hand-dominance	Right	38 (76%)				
	Left	11 (22%)				
Language	Dutch	17 (34%)				
	English	33 (66%)				

Age is presented in years. Educational level is based on completed or expected level of certification. Hand dominance is determined using the Edinburgh Handedness Inventory where values below −40 are interpreted as left-handed, and values greater than +40 are interpreted to be right-handed. Language indicates the preferred language in which the study was conducted.

### 2.2 Measures

#### 2.2.1 SMS task

Participants tapped the index finger of their dominant hand to the beat of the cue on a Novation (High Wycombe, UK) Pro Launchpad (64-pad MIDI controller), in a hammer-like fashion. The auditory conditions were presented in a randomized order, and each lasted 64 beats (the first 4 beats were excluded from analysis for start-up effects). The ST was presented first, followed by the DT of the same condition. Taps were registered in milliseconds using Max7 software (Cycling ’74, Covina, CA, USA). CV indexed tapping variability, calculated as the standard deviation of the inter-tap interval corrected for the mean (range 0–1, with lower scores indicating better consistency). Tapping Force was measured as MIDI velocity on a scale from 0 to 127, presented as a percentage. Trials with < 40 taps were excluded.

For the DT, participants tapped while performing a 2-N-Back working memory task. Here, letters appeared for 0.5 s with 1.5 ± 0.25 s between letters. Participants verbally indicated when the current letter matched the target two letters prior, while instructed to do their best in both tasks. Each trial included 12 letters with 5 correct targets (see Supplementary Figure A1). DTC was calculated by subtracting the dual from the single task performance. For CV, lower values indicate better consistency, thus negative DTC scores indicate greater cost. For Force, lower values indicate reduced force. A positive DTC score indicates Force DT < ST, and a negative DTC indicates Force DT > ST. A DTC of 0 indicates no difference between ST and DT.

#### 2.2.2 Auditory stimuli

Auditory cues, presented through Sony (Tokyo, JP) MDR-ZX110NA headphones, comprised a metronome set at 120 beats per minute (BPM), and a fragment from Genesis by artist Justice (2007, Ed Banger Records/Because Music). This track was selected for its clear and easily detectable beat (117.03 BPM), corresponding closely to the spontaneous motor tempo (McAuley et al., 2006). Both stimuli included 64 beats and were approximately 30 s in duration.

#### 2.2.3 Neuropsychological measures

Participants completed four cognitive measures. The Rey Auditory Verbal Learning Test (RAVLT; Rey, 1941) with a lower score indicating better memory. The Stroop Color-Word Interference Test (Fine and Delis, 2011) measures *inhibitory control* by subtracting the average time in seconds to complete the reading + color (congruent) trials from the incongruent task, where higher values indicate poorer inhibition. The Trail Making Test (TMT; Fine and Delis, 2011) measures *switching ability* by visuo-spatially tracing either only numbers (A) or alternating numbers and letters (B). The score consists of the time needed for TMT-A subtracted from TMT-B (in seconds); lower scores indicate better switching abilities. Finally, the D2 Cancelation Test (Brickenkamp and Zillmer, 2010) measures *sustained attention* and required participants to cross out specific letters in 14 trials of 20 s each. The corrected hit rate is measured by subtracting false positives from total hits, with higher scores indicating better attentional performance.

To measure motor abilities, the Grooved Pegboard Test (GPT; Roy and Square, 1994) indexed fine motor skills, requiring pegs to be placed in a grooved board as fast as possible. Time (seconds) to completion using the dominant hand is measured, where shorter time indicates better fine motor skills. The Box and Blocks Test (BBT; Mathiowetz et al., 1985) measures gross motor function through the total blocks transferred in 60 s using the dominant hand, where higher scores indicate better gross motor function. Participants also completed the Action Research Arm Test (Lyle, 1981), but this was not analyzed as all participants achieved ceiling level.

### 2.3 Procedure

In an online preparatory questionnaire, participants provided informed consent, demographic information (including age, gender, and education level), and completed the Goldsmiths Musical Sophistication Index (GMSI; Müllensiefen et al., 2014), and the Barcelona Music Reward Questionnaire (BMRQ; Mas-Herrero et al., 2014)^2^. In the lab, participants completed 35 min of cognitive measures, followed by 10 min of finger-tapping in a sound-isolated booth. Then, motor measures were administered in another 10–15 min (see Supplementary Figure A2). The cognitive and motor measures were sequenced using Latin Squares counterbalancing, whereas the computer presented the single task first, followed by the dual task and the auditory conditions in a randomized order. This controls for the order of the test presentation within domains, but ensures effects of fatigue are similar across participants, while also keeping the main outcome measures adjacent to the hypothesized predictors. Finally, participants were debriefed and compensated in study credits or €6,50. The entire study lasted approximately 60 min.

### 2.4 Statistical analysis

Analyses were performed in R (R Core Team, 2024); using the “lme4” package (Bates et al., 2015) for linear mixed effects models (LMM) to assess differences in CV and Force between single and dual tasks across both auditory cues. This differs from preregistration as it allows for inclusion of multiple predictors and repeated measures present in the design. Due to non-linear relationships between the neuropsychological measures and tapping outcomes in the ST and DTC, the “mgcv” package for generalized additive models was used (GAMs; Wood, 2017, 2023). This differs from the linear preregistered analyses and includes model estimates and proportion of null deviance explained by the model as relevant outcome indices. Penalized smoothing splines in GAMs accommodated non-linearity while avoiding overfitting. Participants were modeled as a random effect to account for repeated measures. Task, comparing single and dual task conditions, was modeled as fixed effect in LMMs, and Condition, comparing auditory stimulation, was a fixed effect in all models. Finally, to examine whether questionnaires on music reward (BMRQ) and the musical training subscale of the GMSI would alter the results, we performed the same GAM analyses including these questionnaires. Restricted Maximum-Likelihood (REML; Bates et al., 2015; Wood, 2011) was used as the estimation method.

Box-Cox transformations (MASS package; Venables and Ripley, 2002) addressed non-normally distributed residuals in the LMM on CV. The lambda value (−1.19) was chosen through automatic selection (i.e., the λ for the maximum log-likelihood). For the LMM on Force, various transformations (log, square root, cubic, and cubic root) did not address non-normality. However, both LMM and GLMs are robust to violations of distributional assumptions (Coupé, 2018; Schielzeth et al., 2020). Thus, for the LMM on Force and all GAMs, where normality of residuals was violated (visually inspected, and statistically using the Shapiro–Wilk test), sensitivity analyses of the models were conducted. Data points were characterized as residual outliers based on a visual inspection of extremes in a QQplot (approximately ± 2.5 quantiles). If normality of residuals and/or the conclusions of the analysis differed between the analyses with and without outliers, the reported analyses excluded residual outliers, otherwise, the residual outliers were included in the reported model (see Supplementary Material B and E). Multiple comparisons were accounted for using the Bonferroni method, both corrected and uncorrected values are reported (see Supplementary Material B–D, F).

Due to the reported relationships between cognitive and motor abilities, we conducted exploratory GAM analyses to assess whether cognitive measures predicted fine and gross motor function (see Supplementary Material C). We further explored the relationships between tapping Force and CV with cognitive and motor predictors (using similar GAMs analyses as on ST and DTC), but with DT performance (see Supplementary Material D).

## 3 Results

### 3.1 Descriptives of predictors and tapping measures

For participant demographics see Table 1. Table 2 displays tapping outcomes across ST, DT, and DTC, and Table 3 provides descriptives of standardized cognitive, motor, and musical measures. For the exploratory GAMs relating cognitive and motor function, the full sample is included (for results see Supplementary Material C).

**TABLE 2:** Descriptive statistics of timing consistency (CV) and Force of tapping.

		Metronome					Music				
Condition		*N*	*M*	*SD*	Min	Max	*n*	*M*	*SD*	*Min*	*Max*
ST	CV	44	0.07	0.07	0.03	0.35	45	0.05	0.03	0.03	0.25
	Force	45	64.72	30.52	10.48	121.06	45	65.36	30.78	4.47	121.45
DT	CV	44	0.07	0.05	0.03	0.25	45	0.06	0.05	0.03	0.36
	Force	45	67.04	30.52	8.30	120.68	45	66.16	32.38	3.57	121.23
DTC	CV	44	−0.01	0.04	−0.13	0.12	45	−0.01	0.02	−0.11	0.02
	Force	45	−2.3	14.70	−37.13	49.14	45	−0.80	9.60	−26.46	23.64

ST, single task; DT, dual task; DTC, dual task cost; CV, coefficient of variation calculated as the standard deviation of the inter-tap interval in milliseconds corrected for mean with lower scores indicating better consistency; Force measured as MIDI velocity on a scale from 0 to 127 and presented as a percentage, with higher scores indicating more force.

**TABLE 3:** Distribution of scores and ratings of cognitive, motor, and musical measures.

Variable	*n*	*M*	*SD*	*Min*	*Max*
RAVLT	50	1.0	1.5	–2	6
Stroop	50	32.2	12.6	10	68
TMT	49	21.9	16.5	–5	108
D2	50	224.6	42.4	150	303
GPT	50	59.3	8.4	47	86
BBT	50	65.2	9.1	44	87
BMRQ – Total	50	79.2	8.5	55	94
GMSI – MT	50	23.3	10.6	8	48

RAVLT, Rey Auditory Verbal Learning Test calculated as 5^th^ Immediate Trial — Delayed Recall; Stroop Interference calculated as Incongruent — Congruent Trials time in seconds; TMT, Trail Making Test calculated as Switching — Counting Time (Trial B–A) in seconds; D2 calculated as corrected hit rate (correct hits — false positives); GPT, Grooved Pegboard Task calculated as time to complete in seconds; BBT, Box and Blocks Test calculated as total count of transferred blocks in one minute; BMRQ, Barcelona Music Rating Questionnaire — Total, the sum of all subscales; GMSI-MT, Musical Training subscale of the Gold Music Sophistication Index.

### 3.2 Tapping measures across auditory cues and single/dual task

**FIGURE 1:**
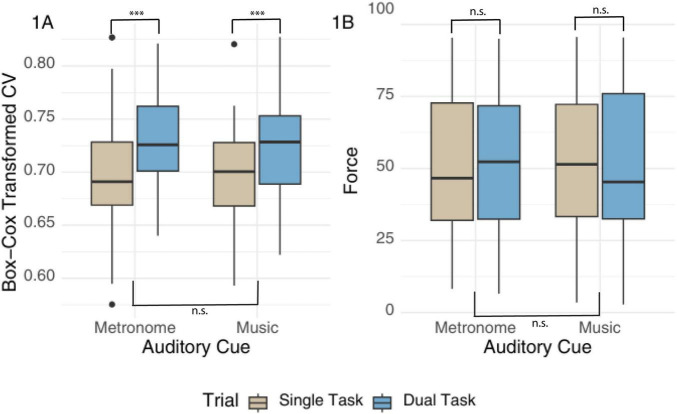
A boxplot of CV and Force by auditory cue and task type. Panel **(A)** Box-Cox transformed values of tapping consistency (CV) measured in milliseconds, and panel **(B)** shows tapping force as a percentage based on MIDI velocity responses. Results are presented by auditory condition and task type. Results are indicated by (***) for *p*-values < 0.001; n.s. indicates non-significant findings.

Results showed a significant effect of task (estimate = 0.03, SE = 0.01, CI [95] 0.01–0.04, *p* < 0.001), with larger CV (lower consistency) during DT than ST across both auditory conditions (see Figure 1A). There were no differences between auditory cues, and no Task-*x*-Auditory Condition interaction. The intraclass coefficient was 0.37, with a significant intercept.

Removing residual outliers resulted in normally distributed residuals when comparing Force between conditions and tasks but did not change the model conclusions. Results without extreme residuals showed no difference in Force between cues, tasks, or their interaction; see Figure 1B. The intercept was significant (estimate = 50.74, SE = 3.65, CI[95] 43.53–57.94, *p* < 0.001). Including music questionnaires did not predict this outcome for both models, but the intercept was no longer significant for the model on Force (estimate = 30.30, SE = 31.35, *p* = 0.39).

### 3.3 Cognitive abilities and tapping outcomes

For tapping consistency, results showed that none of the cognitive measures significantly predicted CV [*R*^2^(adj.) = −0.08, 4.21% deviance explained], but the intercept was significant (see Supplementary Figure B2 and Supplementary Table B4, for DT see Supplementary Table D2).

**FIGURE 2:**
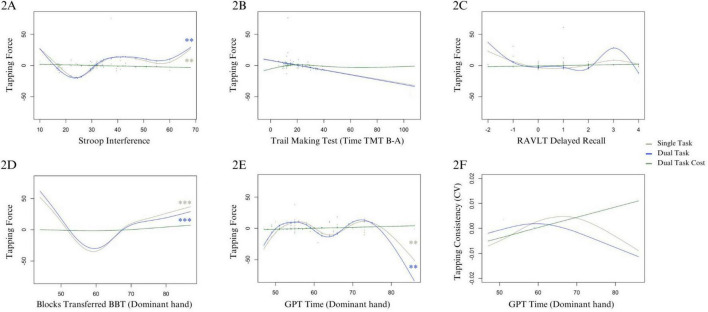
Cognitive and motor predictors of tapping force and consistency for ST, DT, and DTC. Panel **(A)** shows Stroop interference on the *x*-axis (with greater numbers indicating greater interference, i.e., worse performance), and tapping force on the *y*-axis. Panels **(B,C)** show outcomes of exploratory analyses in the DT of TMT and RAVLT on force, respectively. Panel **(D)** shows BBT score, where higher scores indicate better performance, tapping force on the *y*-axis, and panel **(E)** shows GPT time to completion where greater values indicate worse performance. Panel **(F)** shows GPT time to completion for the dominant hand where greater values indicate worse performance, and the *y*-axis indicates CV, where lower values similarly indicate better consistency. For models including residual outliers, the residuals are shown **((A–C,E))**. For models reported without residual outliers, the data points are not shown **(D,F)**. Significant Bonferroni-corrected results are indicated by (**) for *p*-values <0.01, and (***) for *p*-values <0.001.

For Force, residuals were non-normally distributed. Removing residual outliers did not change normality or model conclusions; thus, the reported results include residual outliers. The Durbin–Watson test indicates auto-correlation (DW = 1.41, *p* = 0.002), which is accounted for by the experimental design. For both the ST (edf = 4.80, df = 5.85, *X*^2^ = 16.58, *p* = 0.009) and DT (edf = 4.79, df = 5.83, *X*^2^ = 19.27, *p* = 0.003), Stroop interference non-linearly predicted higher Force at both low and high extremes, except for top performers (i.e., lowest interference < 20) with an upward trend showing interference predicting greater Force (see Figure 2A). The model explained 29.6% [*R*^2^(adj.) = 0.19] and 41% [*R*^2^(adj.) = 0.30] of the deviance for ST and DT, respectively. Intercepts of both models were significant (see Figures 2B, C and Supplementary Table B3).

### 3.4 Motor abilities and tapping outcomes

For CV, residuals were non-normally distributed, however, normality improved when removing residual outliers. As model conclusions differed, results are reported without residual outliers. Only GPT initially predicted CV in ST (edf = 2.42, df = 3.04, *X*^2^ = 7.97, *p* = 0.047), however this effect was no longer significant with extreme residuals removed (edf = 2.38, df = 2.99, *X*^2^ = 7.23, *p* = 0.059). Figure 2F illustrates that for lowest and highest GPT performance, consistency is better than those with middle range of GPT scores. The model explained 6.47% of the deviance [*R*^2^(adj.) = −0.07]. This effect was not observed for DT (edf = 1.82, df = 2.23, *X*^2^ = 2.48, *p* = 0.417). The intercept was significant in both models. See Supplementary Table B6 and Supplementary Figure B4 for ST, and D4 for DT performance.

Both GPT and BBT significantly predicted Force in ST (edf = 5.10, df = 6.08, *X*^2^ = 21.39, *p* = 0.002; edf = 4.85, df = 5.80, *X*^2^ = 64.06, *p* = 0.001), and DT (GPT: edf = 5.25, df = 6.24, *X*^2^ = 19.20, *p* = 0.005; BBT: edf = 4.50, df = 5.40, *X*^2^ = 39.86, *p* < 0.001). Reduced Force was non-linearly predicted by both shorter but especially longer (i.e., worse) GPT times (Figure 2E). Force decreased for BBT scores below 60, and increased with BBT scores 60 and higher (Figure 2D). The intercept was significant (Supplementary Table B5). The model explained 56% of deviance [*R*^2^(adj.) = 0.493] for ST, and 46.3% for DT [*R*^2^(adj.) = 0.384; see Supplementary Material D3].

### 3.5 Cognitive and motor predictors of DTC

Neither cognitive nor motor abilities significantly predicted DTC for CV or Force (*p* > 0.05), with the exception of the models including musical questionnaires (see section 3.6). Auditory cues did not significantly predict DTC in any of the GAMs (see Supplementary Tables B7–10).

### 3.6 Role of musical training and reward

Adding musical reward/training questionnaires as covariates showed a similar pattern of results, with a few exceptions. While the impact of cognitive factors on CV was unchanged, Force was now significantly predicted by the Stroop, RAVLT, and D2 in both ST [edf = 0.85–7.68, df = 1.00–8.34, *X*^2^ = 5.73–57.01, *p* < 0.02; *R*^2^(adj.) = 0.686; deviance explained = 77.7%] and DT [edf = 0.90–8.05, df = 1.00–8.56, *X*^2^ = 9.11–94.74, *p* < 0.001; *R*^2^(adj.) = 0.826; deviance explained = 89.5%]. Notably, the effect sizes are larger after adding music questionnaires only for the DT (*X*^2^ = 9.11–94.74 vs. 2.38–19.27). For motor predictors of CV or Force, results remain consistent in ST, DT, and DTC. In both cognitive and motor domain models, musical training is a significant predictor of CV for both ST/DT, and of Force in DTC only (*p* < 0.001, *X*^2^ = 21.32–27.86), whereas musical reward is only a predictor of Force in the model with cognitive predictors for ST, DT, and DTC (*p* < 0.01, *X*^2^ = 17.50–33.12; see Supplementary Material E).

## 4 Discussion

We investigated whether individual differences in cognitive and motor abilities predicted rhythmic finger tapping (as measured by timing consistency and force) under ST and DT conditions. We used auditory cues with varying rhythmic complexity but high beat clarity (metronome vs. music). The present study results showed that tapping consistency, but not force, declined for DT. Additionally, individual differences in inhibitory control inversely predicted tapping force, with poorer inhibition predicting greater force. Accounting for musical questionnaires additionally revealed that verbal memory, sustained attention and reward from music predicted tapping force. For motor abilities, better gross motor function predicted greater force, whereas those with the poorest fine motor ability applied less force. These non-linear relationships were evident in both ST and DT, but not for DTC with a few exceptions, indicating no specific competition for resources. For DTC, both music reward in the cognition model and fine motor ability showed a positive relation with tapping force, as well as those with the highest musical training similarly applied greater force. Moreover, no tapping differences were observed between the metronome and music conditions. Thus, adding cognitive load only deteriorated SMS timing and not tapping force. While finger tapping to music or a metronome was not different, individuals’ cognitive and motor abilities are shown to predict SMS aspects, where better inhibitory control and fine motor ability predicted lower tapping force, and gross motor function predicted higher tapping force. These non-linear relations were evident irrespective of cognitive load.

### 4.1 Rhythmic complexity and beat clarity

Not identifying an effect of auditory cue complexity on SMS may be related to beat clarity. While beat clarity and rhythmic complexity are conceptually related (Merker et al., 2009; Miguel et al., 2020) both variably influence tapping consistency (Levitin et al., 2018). Although synchronization is often better with lower rhythmic complexity, such as in a metronome (Tranchant et al., 2016), positive effects of music in rhythmic movement are also reported (Rose et al., 2021) among aging (Wittwer et al., 2013) and clinical populations (Rose et al., 2019). We do not find differences between cues with high beat clarity, which may therefore be the most salient cue aspect for this movement type, and accounting for self-reported reward from music and musical training did not affect this finding. Predictably, we found that musical training was associated with better tapping timing consistency for both ST and DT in both models with cognitive and motor predictors. As only one sound fragment per cue was used, this does not preclude differences when varying other features, but it suggests that with consistent beat clarity, similar SMS is observed.

### 4.2 Neuropsychological predictors of SMS

Working memory was previously reported to be implicated in SMS (Colley et al., 2018; Grahn and Schuit, 2012). Additionally, negative autocorrelations of inter-tap intervals were reported for tempo-varying SMS, indicative of a predictive strategy, and attributed to attentional involvement. Moreover, a dual cognitive-motor paradigm led to slowed tapping responses (Guérin et al., 2021), suggesting cognitive involvement. The present finding that tapping consistency was more variable for DT than ST matches these previous observations. Notably, the finding on inhibitory control echoes previous results of it predicting timing variability in healthy participants (Slater et al., 2018) and those with neurocognitive disorders (Löser et al., 2024), establishing the crucial involvement of inhibitory control in SMS (Guérin et al., 2021; Löser et al., 2024). The current findings highlight the relationship of inhibitory control and tapping force irrespective of cognitive load, with lower inhibition ability predicting harder tapping. We attribute this to poorer inhibitory control leading to inefficient motor output, replicating a previous finding (Mudarris and Schaefer, 2022).

Considering motor abilities and SMS, fine motor skills were previously not found to be associated with SMS timing in young adults (Lorås et al., 2013), in contrast to working memory and processing speed. However, the role of cognition in both fine and gross motor skills was investigated using a dual cognitive-motor task, and poorer fine motor skill was associated with DT performance in a fine, but not a gross motor DT (Raisbeck and Diekfuss, 2015). Similarly, the present study found that poorer fine motor ability predicted lower force. Fine motor ability also explained finger tapping variability in children (Monier and Droit-Volet, 2019), further underlining the link between fine motor function and SMS. These findings suggest that motor abilities could influence SMS through these cognitive domains (Guérin et al., 2021). The current results indicate gross motor function (gripping and moving blocks) also predicts SMS force. This could be due to underlying neural commonalities associated with flexing the fingers (Inui and Katsura, 2002) and applying upper-limb strength. While speculative, it may be that the findings with regards to fine motor abilities and inhibitory control result from shared or proximate brain regions as fine motor abilities are located in the medial precentral gyrus (a.k.a. paracentral) of the posterior frontal lobe, which hosts executive functions (including inhibition) more anteriorly. On the other hand, gross motor function is mapped more laterally on the motor strip, which may help explain the dissociation in our findings. Together, these findings elucidate the interactions of motor and cognitive abilities in SMS timing and force.

### 4.3 Cognitive-motor interference in SMS

Matching prior literature (Maes et al., 2015), we observed that a dual cognitive task hindered SMS timing consistency, but not force. Despite finding cognitive and motor predictors of force in ST and DT, with a few exceptions, this was not evident for DTC. This contrasts with different movement types such as walking (Yogev et al., 2008), where better cognitive capacity predicts lower DTC in older adults and patients with neurological disorders. While SMS is not similarly affected here, this may be due to limited neuropsychological variation in the current sample, leading to smaller DTC. Moreover, previous results suggest that the cognitive DT presentation timing relative to the motor task yields exacerbated interference when presented earlier than expected (Langhanns and Mueller, 2020). As DT timing was not manipulated here, greater interference might be induced in this way. Other effects may be seen with the use of the non-dominant hand (Moore et al., 2017; Navarro-López et al., 2024) or in bimanual tasks (Kim and Yoo, 2019).

### 4.4 Limitations and future directions

Prior studies finding associations between cognitive and motor abilities in SMS used the synchronization-continuation paradigm (Löser et al., 2024; Slater et al., 2018; Zalta et al., 2020), where tapping rhythm needs to be maintained after discontinuing the auditory cue, thereby increasing cognitive demands. As the current null findings could relate to a lack of challenge and lower DTC, future studies may address this by increasing task difficulty, through synchronization-continuation paradigm, a more complex motor sequence, and/or increasing cognitive difficulty (e.g., 3 or 4-back task). Moreover, other studies discussed here include tempo-varying cues, thereby increasing cognitive load (Colley et al., 2018; Guérin et al., 2021), whereas the current results can only speak to stable cues with high beat clarity.

### 4.5 Conclusion and implications

Overall, this study highlights the contributions of inhibitory control and both fine and gross motor abilities to SMS timing consistency and force for ST and DT, but largely not for DTC. As music-based interventions are commonly used in cognitive and motor rehabilitation, our findings identify a need to assess baseline abilities of the individuals in considering their capacity to engage in rhythmic auditory synchronization, over and above the choice of auditory cue. Moreover, we show that adding a cognitive load deteriorates motor timing consistency, extending findings of cognitive-motor interference to SMS. These findings further our understanding of auditory-cued movement, the role of individual cognitive and motor abilities, and their implications for music-based interventions, which inherently involve motor, cognitive, and perceptual domains.

## Data Availability

The datasets, and accompanying code scripts, presented in this study can be found in online repositories. The names of the repository/repositories and accession number(s) can be found here: Open Science Framework (OSF), doi: https://doi.org/10.17605/OSF.IO/PFHN3.

## Footnotes

1 While this study focuses on cognitive and motor aspects, other preregistered hypotheses pertain to subjective factors not examined here, as reported in the methods section.

2 In the online survey, participants also chose a song for a third condition: a self-selected cue. After each trial, participants also rated each auditory condition (e.g., familiarity, preference, mood) using a visual slider scale. These outcomes are not a part of the present work.
