# Supplementary material for: Cognitive and motor abilities predict auditory-cued finger tapping in a dual task
Source: Front Neurosci. 2025 May 21;19:1553548. doi: 10.3389/fnins.2025.1553548 (PMC12133802; doi:10.3389/fnins.2025.1553548)
Supplement: Supplementary file 1 [file Data_Sheet_1.pdf]

(A)

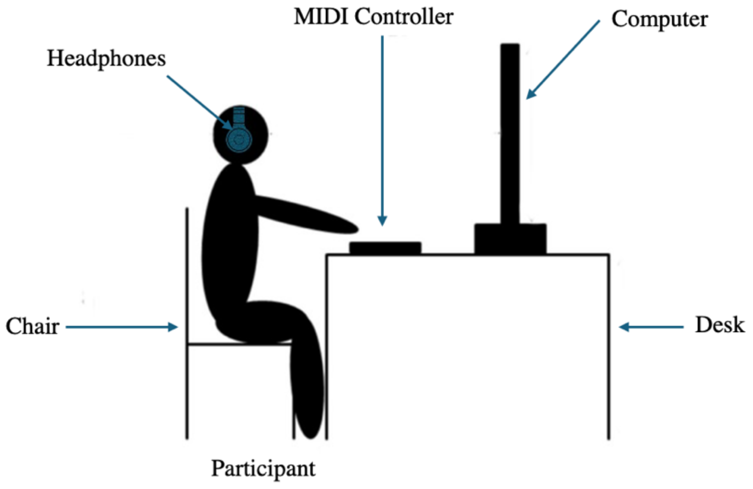

(B)

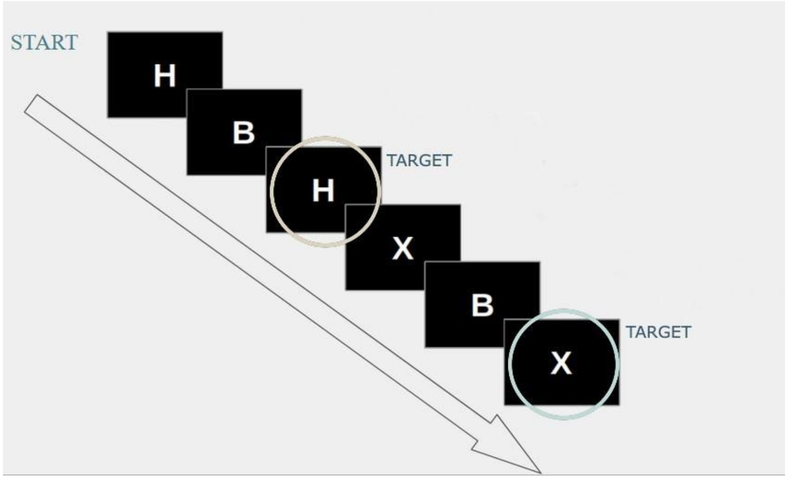

Supplementary Figure A1. The participant is required to sit at a desk with comfortable access to MIDI Controller in clear view of the computer screen (A); An example of the 2 back task in which participants must identify if the current item in a sequence matches the one presented two steps earlier (B).

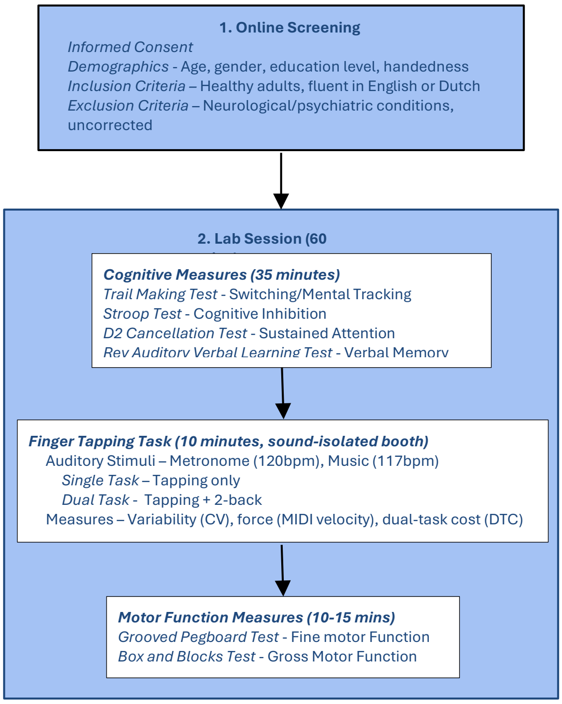

Supplementary Figure A2. The study procedure including online screening, and order of administration during the research visit is depicted.
