# Supplementary material for: Cognitive and motor abilities predict auditory-cued finger tapping in a dual task
Source: Front Neurosci. 2025 May 21;19:1553548. doi: 10.3389/fnins.2025.1553548 (PMC12133802; doi:10.3389/fnins.2025.1553548)
Supplement: Supplementary file 2 [file Data_Sheet_2.pdf]

Supplementary Material B

Results Tables and Figures

Tapping measures across auditory cue type with and without cognitive load

Table B1

Mixed Model Results for %CV by Condition and Trial

	**Box-Cox Transformed CV**		
Predictors	*Estimates*	*std. Error*	*CI*
(Intercept)	0.70 ***	0.01	0.69 – 0.72
Auditory Cue [2]	-0.00	0.01	-0.02 – 0.01
Task [2]	0.03 ***	0.01	0.01 – 0.04
Cue [2] *x* Task [2]	-0.00	0.01	-0.03 – 0.02
**Random Effects**			
σ^2	0.00		
$\tau_{00\ PPT}$	0.00		
ICC	0.37		
N_{PPT}	50		
Observations	178		
Marginal R^2 / Conditional R^2	0.077 / 0.419		

*Bonferroni corrected α-values * p<0.05 ** p<0.01 *** p<0.001*

Table B2

Mixed Model Results for %Force by Condition and Trial

	%Force		
Predictors	*Estimates*	*std. Error*	*CI*
(Intercept)	50.74 ***	3.65	43.53 – 57.94
Auditory Cue [2]	0.73	1.43	-2.11 – 3.56
Task [2]	2.05	1.43	-0.78 – 4.88
Cue [2] *x* Task [2]	-1.42	2.01	-5.40 – 2.55
**Random Effects**			
σ^2	44.95		
$\tau_{00\ PPT}$	551.92		
ICC	0.92		
N_{PPT}	45		
Observations	178		
Marginal R^2 / Conditional R^2	0.001 / 0.925		

*Bonferroni corrected p-values * p<0.05 ** p<0.01 *** p<0.001*

Table B3

Results Summary GAM Single Task Cognition and Tapping Force

Smoothing terms		*Edf*	*df*	χ^2	*p*	*Bonferroni α*
s(RAVLT)		2.96	3.62	4.35	.363	.726
s(Stroop)		4.80	5.85	16.58	.009**	.018*
s(TMT B-A)		1.00	1.00	3.94	.062	.124
s(D2)		1.00	1.00	0.46	.498	.996
s(PPT)		0.61	1.00	1.58	.102	.204
Parametric coefficients	Estimate	*SE*	*z*		*p*	
(Intercept)	69.93	9.91	6.96		< .001 ***	< .001***
Auditory Cue	0.64	5.78	0.11		.911	1.00
R^2 (adj.)	0.193		Deviance explained		29.6%	

Note. RAVLT = Rey Auditory Verbal Learning Test calculated as 5th Immediate Trial Recall – Delayed Recalled Items; Stroop = calculated as Incongruent – Congruent Trials Time in seconds; TMT = Trail Making Test calculated as Switching – Counting Time (B-A) in seconds; D2 calculated as corrected hit rate (correct hits – false positives); PPT = Participants. P-corrected represents Bonferroni corrected p-values.

Figure B1

Partial Effect Plots GAM Single Task Cognition and Tapping Force

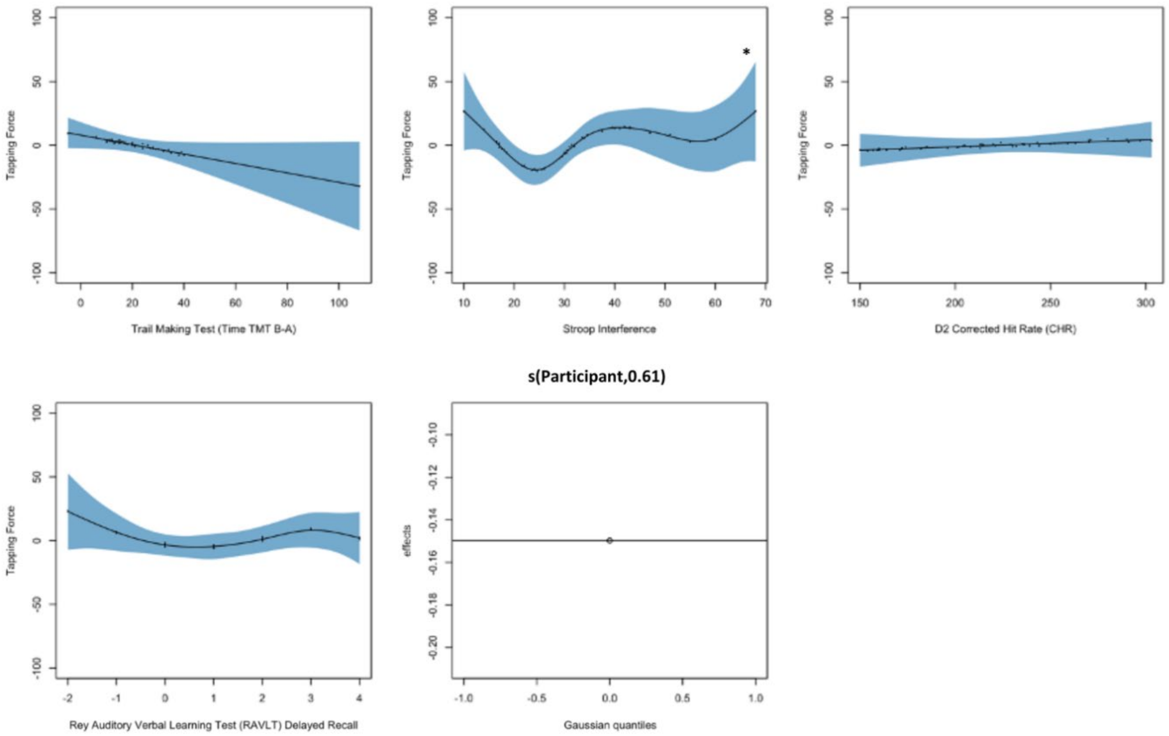

Note. Graphs visualize results with residual outliers included. The solid line represents the fitted relationship, and the shaded area represents the 95% confidence interval of the estimated smooth effect.

Table B4

Results Summary GAM Single Task Cognition and Tapping Consistency

Smoothing terms		*Edf*	*df*	χ^2	*p*	*Bonferroni α*
s(RAVLT)		1.34		1.03	.375	.750
s(Stroop)		1.54		2.06	.415	.830
s(TMT B-A)		1.39		0.85	.653	1.00
s(D2)		1.00		0.07	.792	1.00
s(PPT)		$4.59\text{x}10^{-6}$		0.00	.834	1.00
Parametric coefficients	Estimate	*SE*	*z*		*p*	
(Intercept)	0.041	0.004	8.58		< .001 ***	< 0.001***
Auditory Cue	0.003	0.003	0.85		.394	.788
R^2 (adj.)	-0.083		Deviance explained		4.21%	

Note. Results with residual outliers included. RAVLT = Rey Auditory Verbal Learning Test calculated as 5th Immediate Trial Recall – Delayed Recalled Items; Stroop = calculated as Incongruent – Congruent Trials Time in seconds; TMT = Trail Making Test calculated as Switching – Counting Time (B-A) in seconds; D2 calculated as corrected hit rate (correct hits – false positives); PPT = Participants.

Figure B2

Partial Effect Plots GAM Single Task Cognition and Tapping Consistency

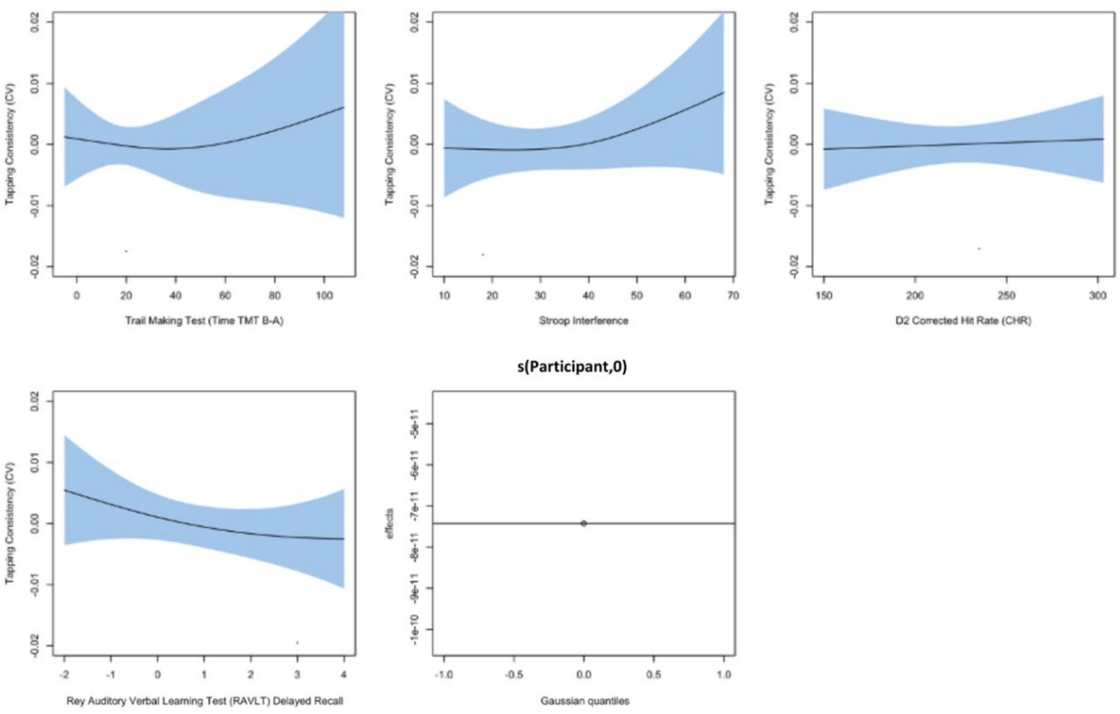

Note. Graphs visualize results with residual outliers included. The solid line represents the fitted relationship, and the shaded area represents the 95% confidence interval of the estimated smooth effect.

Table B5

Results Summary GAM Single Task Motor Ability and Tapping Force

Smoothing terms		*Edf*	*df*	χ^2	*p*	*Bonferroni α*
s(GPT)		5.10		21.39	.002 **	.004 **
s(BBT)		4.85		64.06	< .001 ***	< .001 ***
s(PPT)		0.78		3.48	.031*	.062
Parametric coefficients	Estimate	*SE*	*z*		*p*	
(Intercept)	70.57	8.03	8.79		< .001 ***	< .001 ***
Auditory Cue	0.64	4.58	0.14		.888	1.00
R^2 (adj.)	0.493		Deviance explained		56%	

Note. Results with residual outliers included. GPT = Grooved Pegboard Task calculated as time to complete in seconds; BBT= Box and Blocks Test calculated as total count of transferred blocks; PPT = Participants.

Figure B3

Partial Effect Plots GAM Single Task Motor Ability and Tapping Force

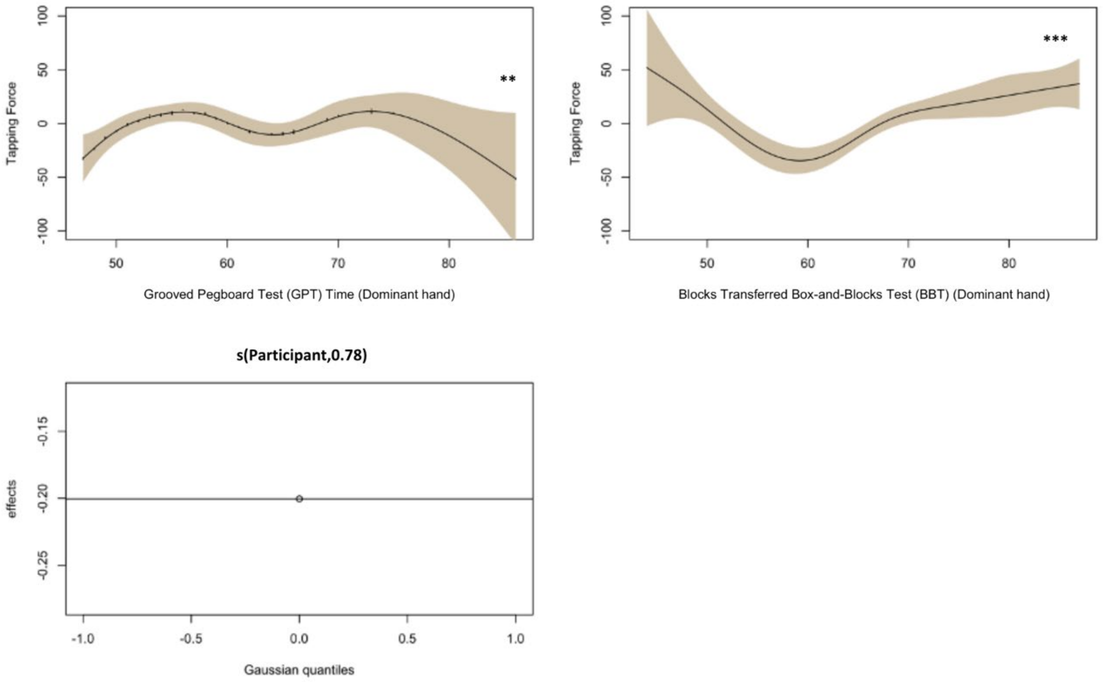

Note. Graphs visualize results with residual outliers included. The solid line represents the fitted relationship, and the shaded area represents the 95% confidence interval of the estimated smooth effect.

Table B6

Results Summary GAM Single Task Motor Ability and Tapping Consistency

Smoothing terms		*Edf*	*df*	χ^2	*p*	*Bonferroni α*
s(GPT)		2.42	2.99	7.23	.060	.120
s(BBT)		1.00	1.00	1.19	.276	.552
s(PPT)		0.07	1.00	0.08	.279	.558
Parametric coefficients	Estimate	*SE*	*z*		*p*	
(Intercept)	0.043	0.005	8.99		< .001 ***	< .001 ***
Auditory Cue	0.001	0.003	0.45		.65	1.00
R^2 (adj.)	-0.068		Deviance explained		6.47%	

Note. Results with residual outliers removed. GPT = Grooved Pegboard Task calculated as time to complete in seconds; BBT= Box and Blocks Test calculated as total count of transferred blocks; PPT = Participants.

Figure B4

Partial Effect Plots GAM Single Task Motor Ability and Tapping Consistency

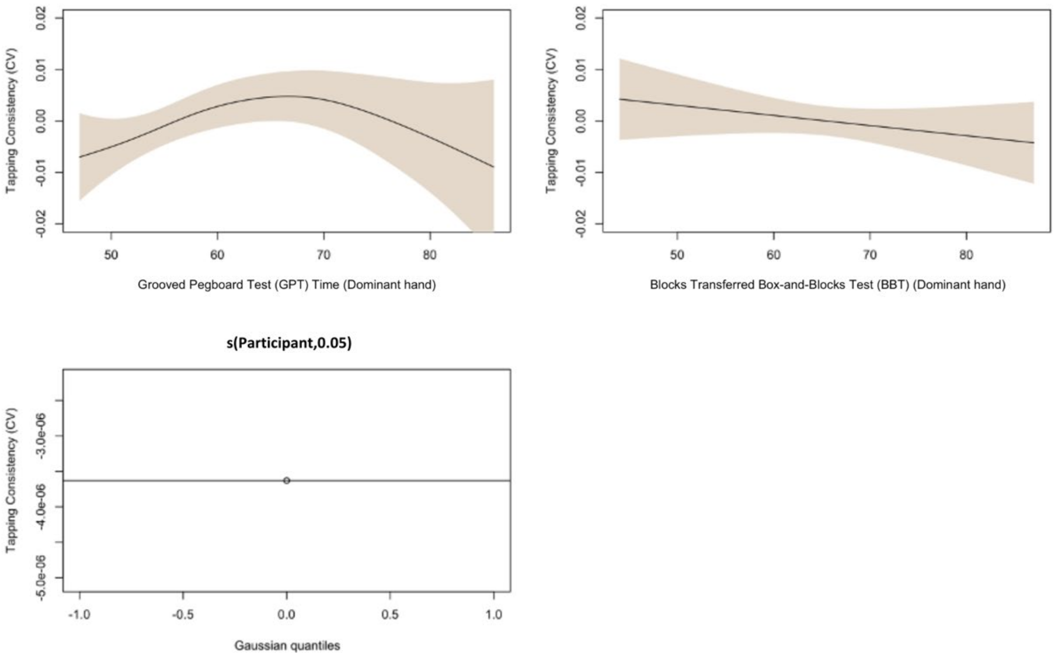

Note. Graphs visualize results with residual outliers included. The solid line represents the fitted relationship, and the shaded area represents the 95% confidence interval of the estimated smooth effect.

DTC – Cognition and Tapping Force

Table B7

Results Summary GAM Dual Task Cognition and Tapping Force

Smoothing terms		*Edf*	*df*	χ^2	*p*	*Bonferroni α*
s(RAVLT)		1.00	1.00	1.31	.252	.504
s(Stroop)		1.00	1.00	1.04	.307	.614
s(TMT B-A)		2.72	3.26	4.07	.298	.596
s(D2)		1.76	2.17	1.92	.406	.812
s(PPT)		0.510	1.00	1.15	.131	.626
Parametric coefficients	Estimate	*SE*	*z*		*p*	.
(Intercept)	-4.21	3.27	-1.29		.197	.394
Auditory Cue	2.29	1.92	1.19		.235	.470
R^2 (adj.)	0.031		Deviance explained		8.81%	

Note. Results with residual outliers included. RAVLT = Rey Auditory Verbal Learning Test calculated as 5th Immediate Trial Recall – Delayed Recalled Items; Stroop = calculated as Incongruent – Congruent Trials Time in seconds; TMT = Trail Making Test calculated as Switching – Counting Time (B-A) in seconds; D2 calculated as corrected hit rate (correct hits – false positives); PPT = Participants.

Figure B5

Partial Effect Plots GAM Dual Task Cognition and Tapping Force

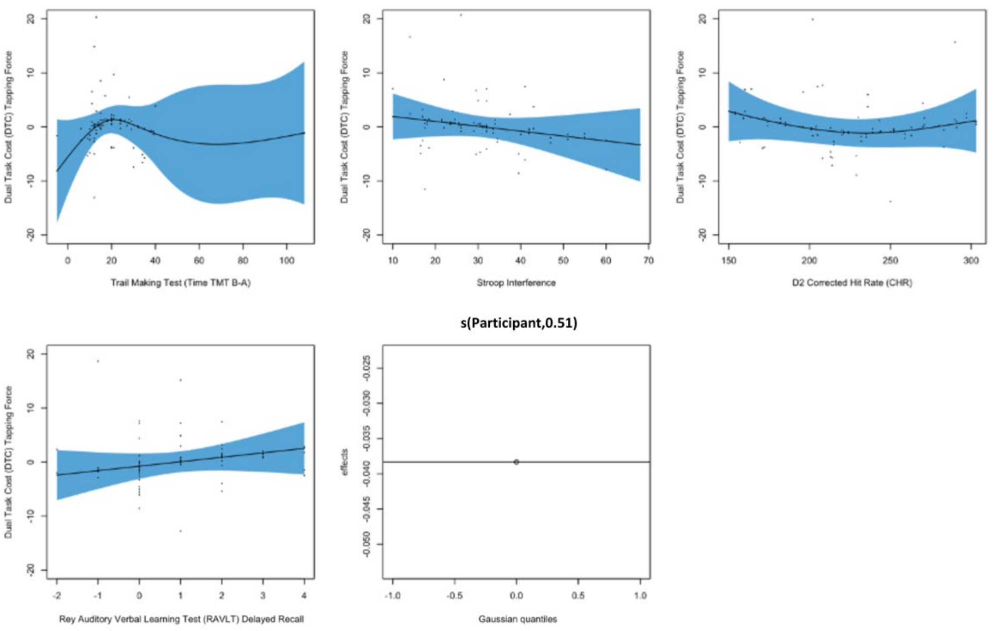

Note. Graphs visualize results with residual outliers included. The solid line represents the fitted relationship, and the shaded area represents the 95% confidence interval of the estimated smooth effect.

DTC – Cognition and Tapping Consistency

Table B8

Results Summary GAM Dual Task Cognition and Tapping Consistency

Smoothing terms		*Edf*	*df*	χ^2	*p*	*Bonferroni α*
s(RAVLT)		1.51	1.87	0.73	.624	1.00
s(Stroop)		1.00	1.00	0.93	.334	.668
s(TMT B-A)		2.76	1.31	5.30	.157	.314
s(D2)		1.00	1.00	0.39	.532	1.00
s(PPT)		3.47×10^{-5}	1.00	0.00	.671	1.00
Parametric coefficients	Estimate	*SE*	*z*		*p*	
(Intercept)	-0.012	0.006	-2.03		.043*	.086
Auditory Cue	0.002	0.004	0.50		.616	1.00
R^2 (adj.)	-0.047		Deviance explained		6.18%	

Note. Results with residual outliers included. RAVLT = Rey Auditory Verbal Learning Test calculated as 5th Immediate Trial Recall – Delayed Recalled Items; Stroop = calculated as Incongruent – Congruent Trials Time in seconds; TMT = Trail Making Test calculated as Switching – Counting Time (B-A) in seconds; D2 calculated as corrected hit rate (correct hits – false positives); PPT = Participants.

Figure B6

Partial Effect Plots GAM Dual Task Cognition and Tapping Consistency

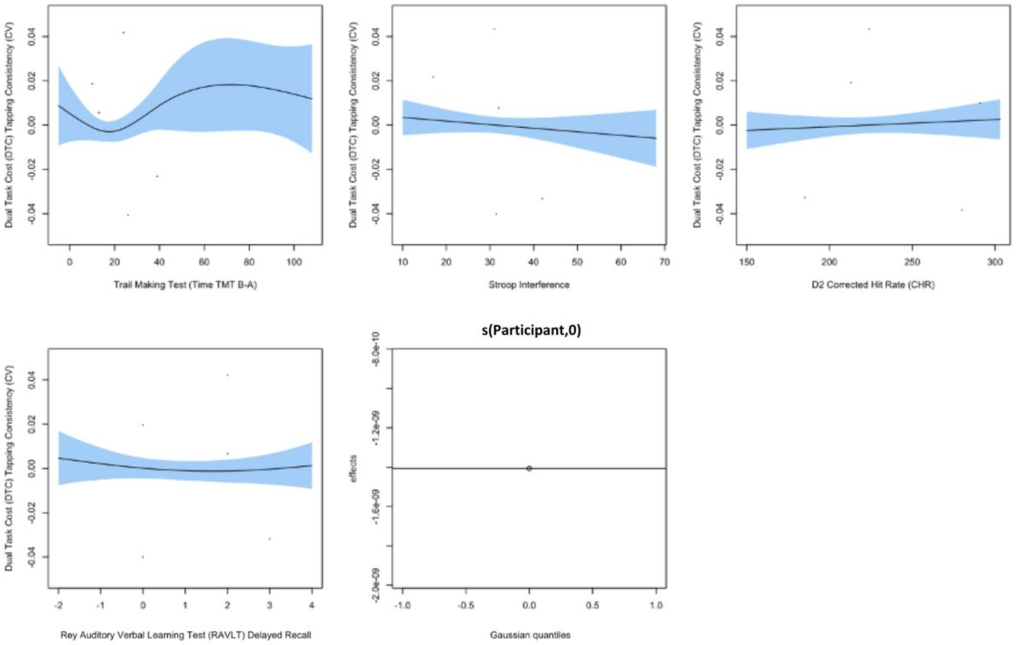

Note. Graphs visualize results with residual outliers included. The solid line represents the fitted relationship, and the shaded area represents the 95% confidence interval of the estimated smooth effect.

DTC – Motor Ability and Tapping Force

Table B9

Results Summary GAM Dual Task Motor Ability and Tapping Force

Smoothing terms		*Edf*	*df*	χ^2	*p*	*Bonferroni α*
s(GPT)		1.00	1.01	1.61	.206	.412
s(BBT)		1.99	2.48	4.77	.110	.220
s(PPT)		2.02×10^{-5}	1.00	0.00	.565	1.00
Parametric coefficients	Estimate	*SE*	*z*		*p*	
(Intercept)	-5.70	2.94	-1.94		.052	.104
Auditory Cue	2.63	1.86	1.42		.157	.314
R^2 (adj.) 0.036			Deviance explained		6.94%	

Note. Results with residual outliers included. GPT = Grooved Pegboard Task calculated as time to complete in seconds; BBT= Box and Blocks Test calculated as total count of transferred blocks; PPT = Participants.

Figure B7

Partial Effect Plots GAM Dual Task Motor Ability and Tapping Force

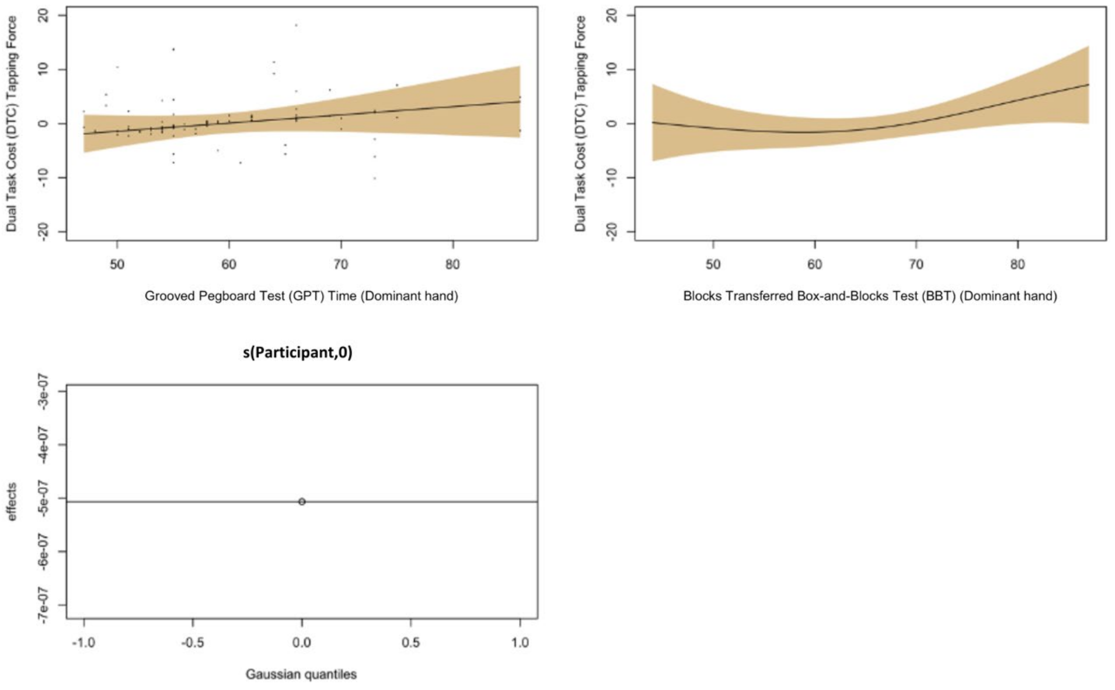

Note. Graphs visualize results with residual outliers included. The solid line represents the fitted relationship, and the shaded area represents the 95% confidence interval of the estimated smooth effect.

DTC – Motor Ability and Tapping Consistency

Table B10

Results Summary GAM Dual Task Motor Ability and Tapping Consistency

Smoothing terms		*Edf*	*df*	χ^2	*p*	*Bonferroni α*
s(GPT)		1.00	1.00	2.88	.090	.180
s(BBT)		1.00	1.00	1.80	.179	.358
s(PPT)		3.43×10^{-6}	1.00	0.00	.745	1.00
Parametric coefficients	Estimate	*SE*	*z*		*p*	
(Intercept)	-0.001	0.01	-1.82		.070	.140
Auditory Cue	0.00	0.003	0.29		.771	1.00
R^2 (adj.)	0.035		Deviance explained		2.9%	

Note. Results with residual outliers included. GPT = Grooved Pegboard Task calculated as time to complete in seconds; BBT= Box and Blocks Test calculated as total count of transferred blocks; PPT = Participant

Figure B8

Partial Effect Plots GAM Dual Task Motor Ability and Tapping Consistency

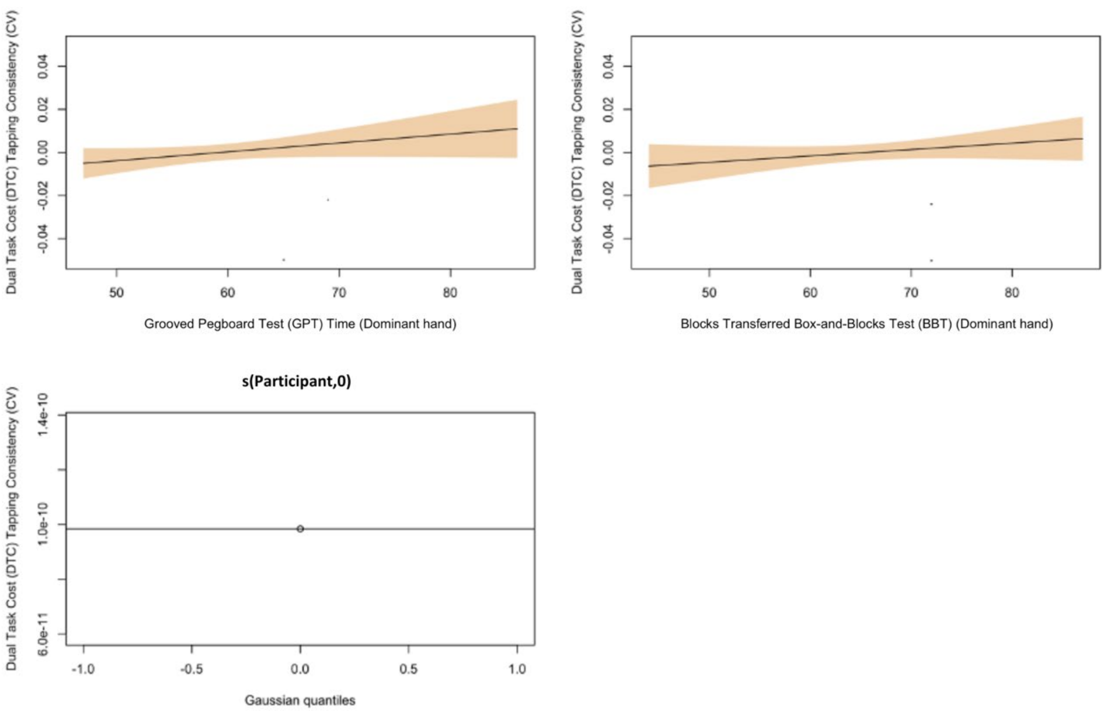

Note. Graphs visualize results with residual outliers included. The solid line represents the fitted relationship, and the shaded area represents the 95% confidence interval of the estimated smooth effect.
