# Supplementary material for: Cognitive and motor abilities predict auditory-cued finger tapping in a dual task
Source: Front Neurosci. 2025 May 21;19:1553548. doi: 10.3389/fnins.2025.1553548 (PMC12133802; doi:10.3389/fnins.2025.1553548)
Supplement: Supplementary file 3 [file Data_Sheet_3.pdf]

Supplementary Material C

Cognitive and Motor Relationships

Cognition and Fine Motor Ability

Residuals are normally distributed after removing extreme residuals. The Durbin-Watson test indicates auto-correlation (0.76, $p < 0.001$), accounted for by the experimental design. RAVLT predicted GPT performance (edf =3.26, df = 3.979, $X^2 = 22.63$, p < .001), with worse delayed recall associated slower GPT completion (Figure 11). The intercept is significant. The model explains 38.7% of deviance (R^2(*adj.)*=0.31). See Table C1.

Table C1. *Results Summary GAM Cognitive Predictors and Fine Motor Ability*

Smoothing terms		*Edf*	*df*	χ^2	*p*	*Bonferroni α*
s(RAVLT)		3.26	3.98	22.64	< .001 ***	< .001 ***
s(Stroop)		1.00	1.00	1.66	.197	.394
s(TMT B-A)		4.01	4.74	8.91	.067	.134
s(D2)		2.06	2.51	5.78	.069	.138
Parametric coefficients	Estimate	*SE*	*z*		*p*	*Bonferroni α*
(Intercept)	59.02	0.70	83.76		< .001 ***	< .001 ***
R^2 (adj.)	0.312		Deviance explained		38.70%	

Note. Results with residual outliers removed. Signif. codes: '***' 0.001 '**' 0.01 '*' 0.05. RAVLT = Rey Auditory Verbal Learning Test calculated as 5th Immediate Trial Recall – Delayed Recalled Items; Stroop = calculated as Incongruent – Congruent Trials Time in seconds; TMT = Trail Making Test calculated as Switching – Counting Time (B-A) in seconds; D2 calculated as corrected hit rate (correct hits – false positives).

Formula:
GPT_TIME_DH ~ s(TMT_B_A_Time) + s(STROOP_CWI, k = -1) + s(D2_CHR, k = -1) + s(RAVLT_T5_DL, k = 7)

Figure C1. *Partial Effect Plots GAM Cognitive Predictors of Fine Motor Function*

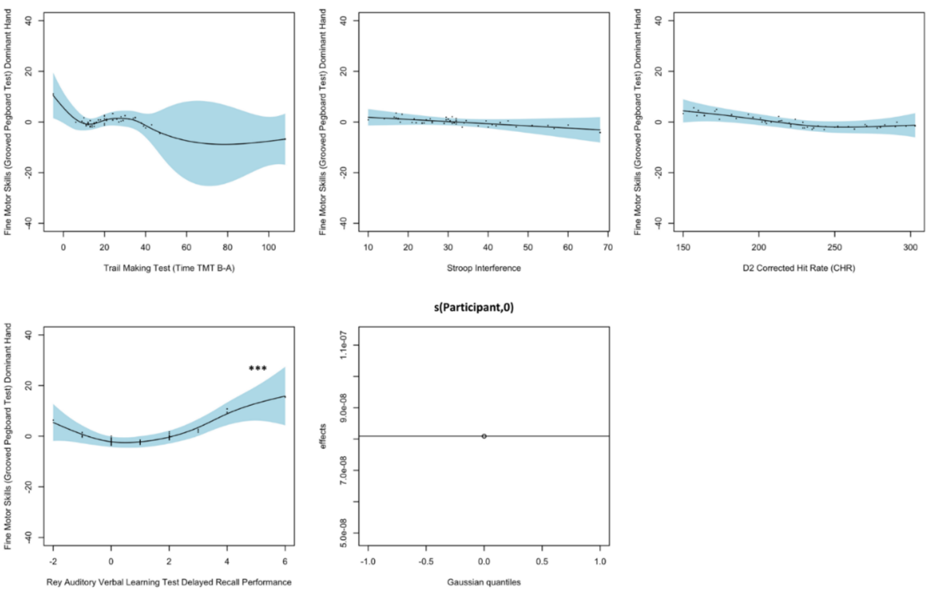

Note. Graphs visualize results when residual outliers are removed. The solid line represents the fitted relationship, and the shaded area represents the 95% confidence interval of the estimated smooth effect.

Cognition and Gross Motor Ability

Stroop interference (edf =4.41, df = 5.41, $X^2 =$ 13.06, p = .03), D2 (edf =7.78, df = 8.57, $X^2 =$ 71.60, p < .001) and RAVLT (edf =1.00, df = 1.00, $X^2 =$ 14.12, p < .001) predicted BBT (R^2(*adj.)*=0.45, 45.6% deviance explained). Greater Stroop interference and poorer RAVLT recall reduced BBT performance, better D2 performance reduced BBT performance. The intercept was significant. See Figure C2 and Table C2.

Table C2

Results Summary GAM Cognitive Predictors of Gross Motor Function

Smoothing terms		*Edf*	*df*	χ^2	*p*	*Bonferroni α*
s(RAVLT)		1.00	1.00	14.13	< .001 ***	< .001 ***
s(Stroop)		4.41	5.41	13.06	.028 *	.056
s(TMT B-A)		1.00	1.000	1.20	.273	.546
s(D2)		7.78	8.57	71.60	< .001 ***	< .001 ***
Parametric coefficients	Estimate	*SE*	*z*		*p*	*Bonferroni α*
(Intercept)	65.15	0.60	108.4		< .001 ***	< .001 ***
R^2 (adj.)	0.446		Deviance explained		45.90%	

Note. Results with residual outliers included. Signif. codes: 0 '***' 0.001 '**' 0.01 '*' 0.05. RAVLT = Rey Auditory Verbal Learning Test calculated as 5th Immediate Trial Recall – Delayed Recalled Items; Stroop = calculated as Incongruent – Congruent Trials Time in seconds; TMT = Trail Making Test calculated as Switching – Counting Time (B-A) in seconds; D2 calculated as corrected hit rate (correct hits – false positives).

Formula:
BBT_DH_COUNT ~ s(TMT_B_A_Time) + s(STROOP_CWI, k = -1) + s(D2_CHR,

k = -1) + s(RAVLT_T5_DL, k = 7)

Figure C2

Partial Effect Plots GAM Cognitive Predictors of Gross Motor Function

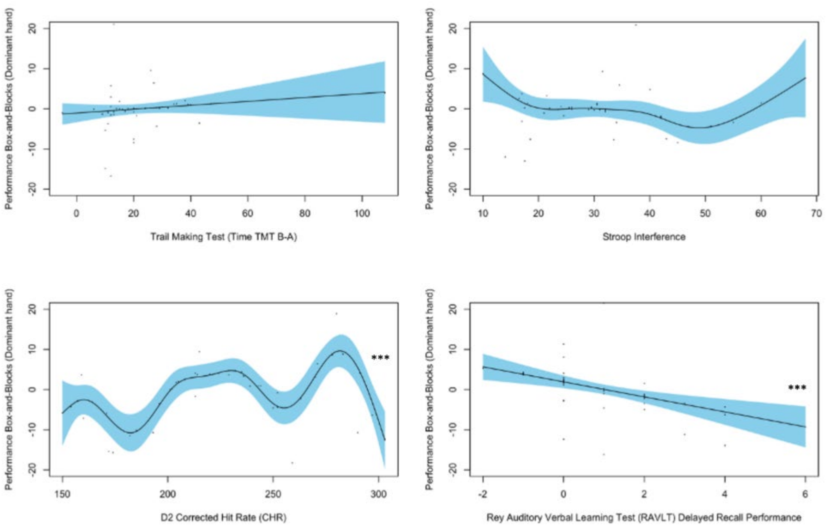

Note. Graphs visualize results with residual outliers included. The solid line represents the fitted relationship, and the shaded area represents the 95% confidence interval of the estimated smooth effect.
