# Supplementary material for: Cognitive and motor abilities predict auditory-cued finger tapping in a dual task
Source: Front Neurosci. 2025 May 21;19:1553548. doi: 10.3389/fnins.2025.1553548 (PMC12133802; doi:10.3389/fnins.2025.1553548)
Supplement: Supplementary file 4 [file Data_Sheet_4.pdf]

Supplementary Material D

GAMs Dual Task Performance

Cognitive Predictors and Tapping Force in the Dual Task

Table D1. *Results Summary GAM Cognitive Predictors and Tapping Force in the Dual Task*

Smoothing terms		*Edf*	*df*	χ^2	*p*	*Bonferroni α*
s(RAVLT)		5.02	5.65	12.24	.025*	.050
s(Stroop)		4.79	5.83	19.27	.003**	.006**
s(TMT B-A)		1.00	1.00	4.09	.043*	.086
s(D2)		1.00	1.00	2.39	.122	.244
s(PPT)		0.71	1.00	2.48	.055	.110
Parametric coefficients	Estimate	*SE*	*z*		*p*	*Bonferroni α*
(Intercept)	74.50	9.65	7.72		< .001 ***	< .001 ***
Auditory Cue	0.89	5.50	-0.16		0.872	1.00
R^2 (adj.)	0.304		Deviance explained		41%	

Note. Results with residual outliers included. Signif. codes: '***' 0.001 '**' 0.01 '*' 0.05. RAVLT = Rey Auditory Verbal Learning Test calculated as 5th Immediate Trial Recall – Delayed Recalled Items; Stroop = calculated as Incongruent – Congruent Trials Time in seconds; TMT = Trail Making Test calculated as Switching – Counting Time (B-A) in seconds; D2 calculated as corrected hit rate (correct hits – false positives); PPT = Participant.

Formula:

FORCE_DT ~ s(TMT_B_A_Time, k = -1) + s(STROOP_CWI, k = -1) +

s(D2_CHR, k = -1) + s(RAVLT_T5_DL, k = 7) + Condition + s(PPT,

bs = "re")

Figure D1. *Partial Effect Plots GAM Cognitive Predictors and Tapping Force in the Dual Task*

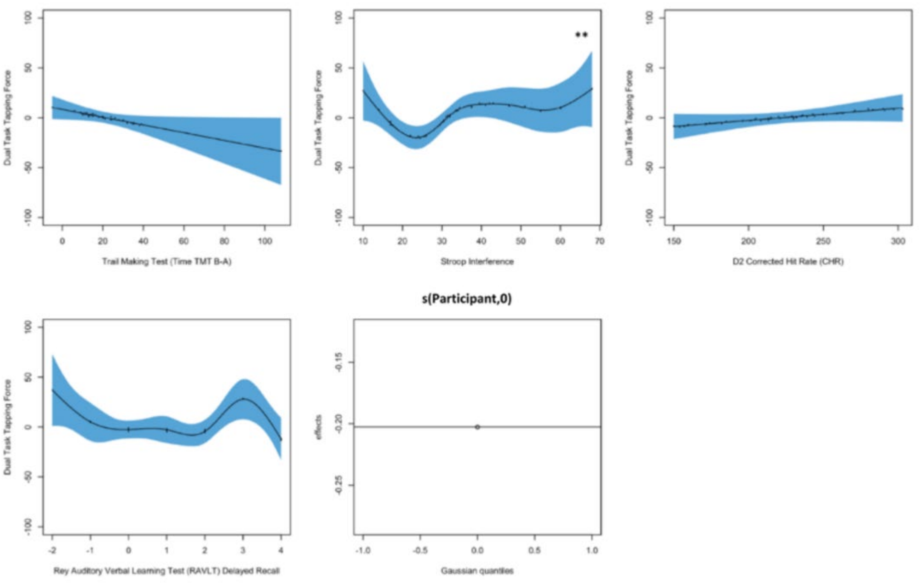

Note. Graphs visualize results when residual outliers are included. The solid line represents the fitted relationship, and the shaded area represents the 95% confidence interval of the estimated smooth effect.

Cognitive Predictors and Tapping Consistency in the Dual Task

Table D2. *Results Summary GAM Cognitive Predictors and Tapping Consistency in the Dual Task*

Smoothing terms		*Edf*	*df*	χ^2	*p*	*Bonferroni α*
s(RAVLT)		1.00	1.00	0.08	.775	1.00
s(Stroop)		1.64	2.04	4.84	.090	.180
s(TMT B-A)		3.01	3.65	8.81	.050	.100
s(D2)		1.00	1.00	0.01	.931	1.00
s(PPT)		$2.12x10^{-5}$	1.00	0.00	.464	.928
Parametric coefficients	Estimate	*SE*	*z*		*p*	*Bonferroni α*
(Intercept)	0.56	0.006	9.87		< .001 ***	< .001 ***
Auditory Cue	-0.00	0.004	-0.213		.832	1.00
R^2 (adj.)	-0.059		Deviance explained		10.9%	

Note. Results with residual outliers included. Signif. codes: '***' 0.001 '**' 0.01 '*' 0.05. RAVLT = Rey Auditory Verbal Learning Test calculated as 5th Immediate Trial Recall – Delayed Recalled Items; Stroop = calculated as Incongruent – Congruent Trials Time in seconds; TMT = Trail Making Test calculated as Switching – Counting Time (B-A) in seconds; D2 calculated as corrected hit rate (correct hits – false positives); PPT = Participant.

Formula:

CV_DT ~ s(TMT_B_A_Time, k = -1) + s(STROOP_CWI, k = -1) + s(D2_CHR, k = -1) + s(RAVLT_T5_DL, k = 7) + Condition + s(PPT, bs = "re")

Figure D2. *Partial Effect Plots GAM Cognitive Predictors and Tapping Consistency in the Dual Task*

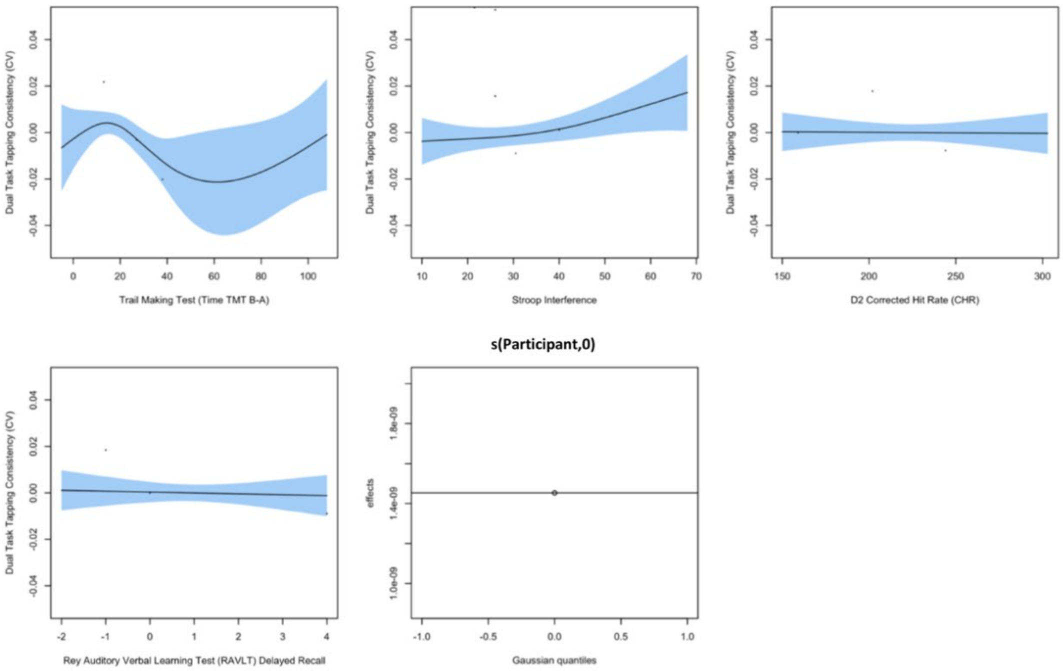

Note. Graphs visualize results when residual outliers are included. The solid line represents the fitted relationship, and the shaded area represents the 95% confidence interval of the estimated smooth effect.

Motor Predictors and Tapping Force in the Dual Task

Table D3. *Results Summary GAM Motor Predictors and Tapping Force in the Dual Task*

Smoothing terms		*Edf*	*df*	χ^2	*p*	*Bonferroni α*
s(GPT)		5.25	6.24	19.20	.005 **	.010 **
s(BBT)		4.50	5.40	39.86	< .001 ***	< .001 ***
s(PPT)		0.65	1.00	1.84	.085	.170
**Parametric coefficients**	**Estimate**	***SE***	***z***		***p***	***Bonferroni α***
(Intercept)	72.79	8.93	8.15		< .001 ***	< .001 ***
Auditory Cue	-0.89	5.18	-0.17		.864	1.00
R^2 (adj.) 0.384			Deviance explained		46.3%	

Note. Results with residual outliers included. Signif. codes: '***' 0.001 '**' 0.01 '*' 0.05. GPT = Grooved Pegboard Task calculated as time to complete in seconds; BBT= Box and Blocks Test calculated as total count of transferred blocks; PPT = Participants.

Formula:

FORCE_DT ~ s(GPT_TIME_DH, k = -1) + s(BBT_DH_COUNT, k = -1) +

Condition + s(PPT, bs = "re")

Figure D3. *Partial Effect Plots GAM Motor Predictors and Tapping Force in the Dual Task*

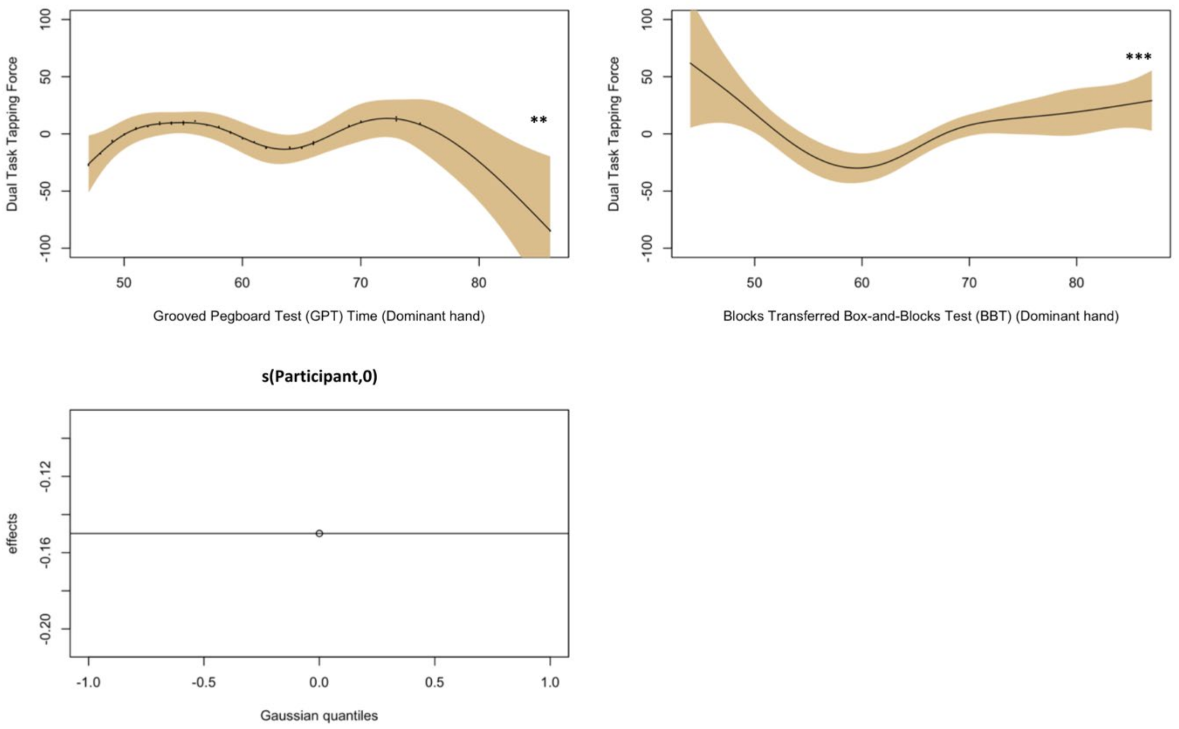

Note. Graphs visualize results when residual outliers are included. The solid line represents the fitted relationship, and the shaded area represents the 95% confidence interval of the estimated smooth effect.

Motor Predictors and Tapping Consistency in the Dual Task

Table D4. *Results Summary GAM Motor Predictors and Tapping Consistency in the Dual Task*

Smoothing terms		*Edf*	*df*	χ^2	*p*	*Bonferroni α*
s(GPT)		1.82	2.30	2.48	.417	.834
s(BBT)		1.00	1.00	2.31	.128	.256
s(PPT)		3.79×10^{-6}	1.00	0.00	.570	1.00
**Parametric coefficients**	**Estimate**	***SE***	***z***		***p***	***Bonferroni α***
(Intercept)	0.06	0.01	9.10		< .001 ***	< .001 ***
Auditory Cue	-0.00	0.00	-0.07		.943	1.00
R^2 (adj.) -0.040		Deviance explained			2.78%	

Note. Results with residual outliers included. Signif. codes: '***' 0.001 '**' 0.01 '*' 0.05. GPT = Grooved Pegboard Task calculated as time to complete in seconds; BBT= Box and Blocks Test calculated as total count of transferred blocks; PPT = Participants.

Formula:
CV_DT ~ s(GPT_TIME_DH, k = -1) + s(BBT_DH_COUNT, k = -1) + Condition +
s(PPT, bs = "re")

Figure D4. *Partial Effect Plots GAM Motor Predictors and Tapping Consistency in the Dual Task*

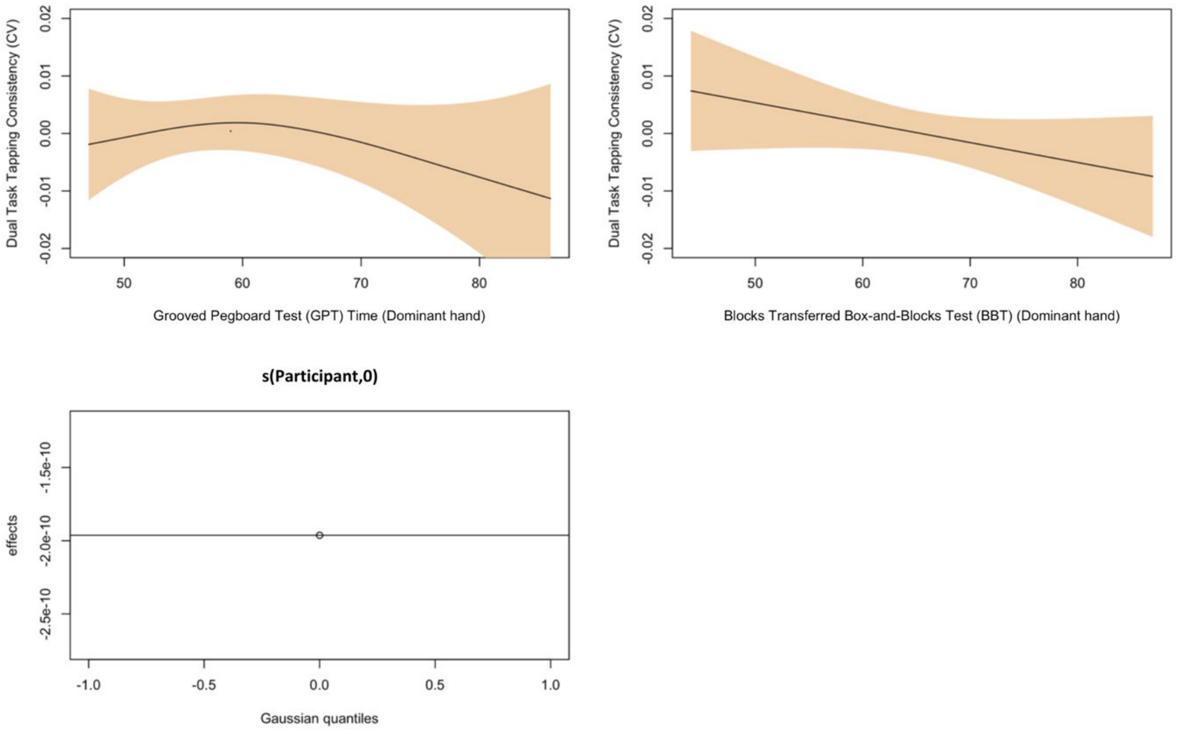

Note. Graphs visualize results when residual outliers are included. The solid line represents the fitted relationship, and the shaded area represents the 95% confidence interval of the estimated smooth effect.
