# Supplementary material for: Cognitive and motor abilities predict auditory-cued finger tapping in a dual task
Source: Front Neurosci. 2025 May 21;19:1553548. doi: 10.3389/fnins.2025.1553548 (PMC12133802; doi:10.3389/fnins.2025.1553548)
Supplement: Supplementary file 5 [file Data_Sheet_5.pdf]

Supplementary Material E

Residual Plots of Main Analyses

Cognitive Predictors of Tapping Force for the Single Task

Figure E1. Q-Q Plot of Residuals of the model with cognitive predictors and tapping force for the single task.

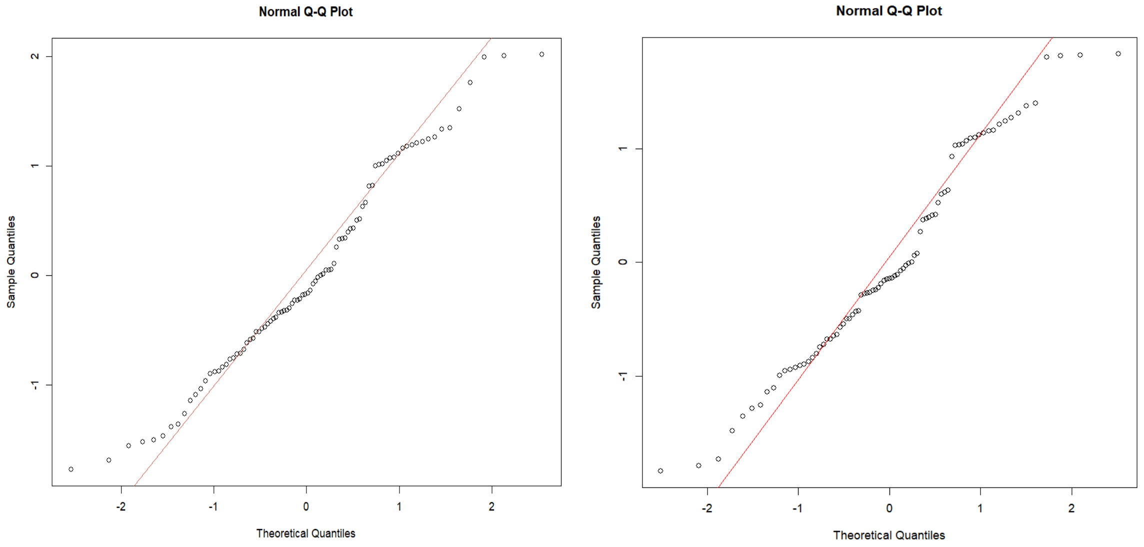

Note. QQ-plot of residuals from the model of single task tapping force with cognitive predictors including extreme residuals (left), and with extreme residuals removed (right).

Cognitive Predictors of Tapping Consistency for the Single Task

Figure E2. Q-Q Plot of Residuals of the model with cognitive predictors of tapping consistency for the single task.

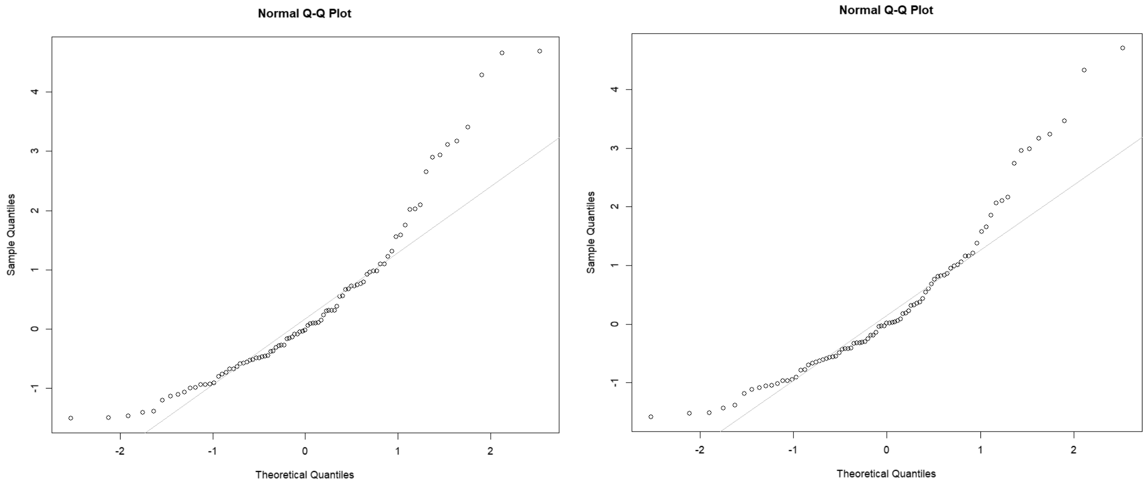

Note. QQ-plot of residuals from the model of single task tapping consistency with cognitive predictors, including extreme residuals (left), and with extreme residuals removed (right).

Motor Predictors of Tapping Force for the Single Task

Figure E3. Q-Q Plot of Residuals of the model with motor predictors of tapping force for the single task.

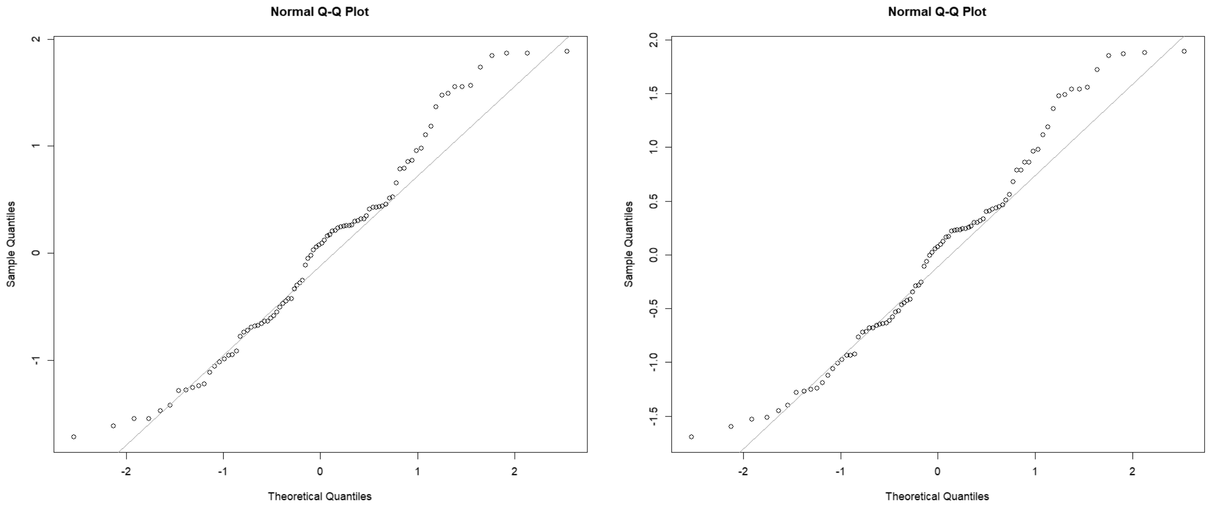

Note. QQ-plot of residuals from the model of single task tapping force with motor predictors, including extreme residuals (left), and with extreme residuals removed (right).

Motor Predictors of Tapping Consistency for the Single Task

Figure E4. Q-Q Plot of Residuals of the model with motor predictors of tapping consistency for the single task.

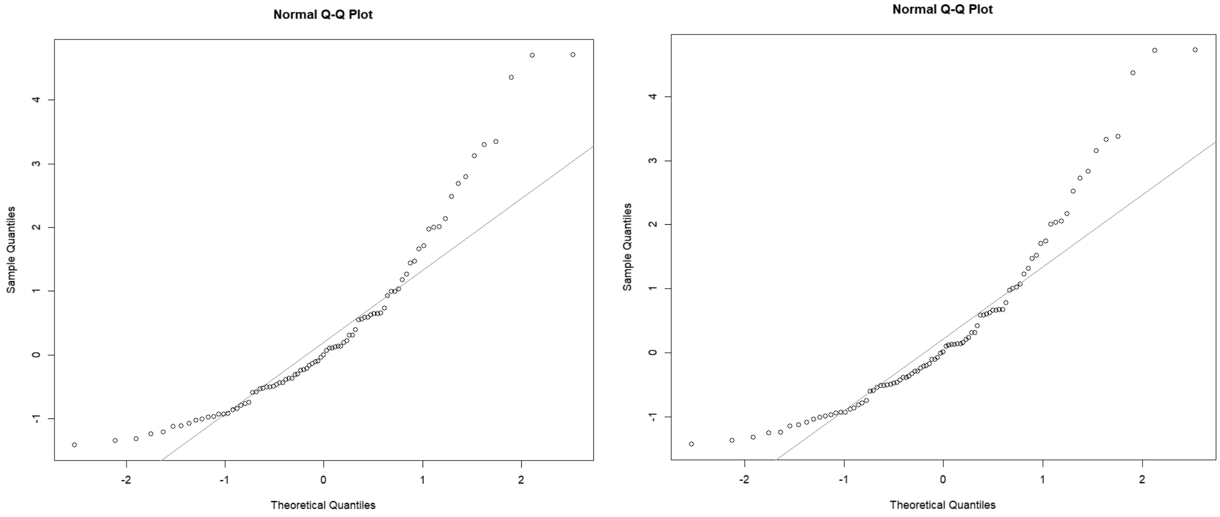

Note. QQ-plot of residuals from the model of single task tapping consistency with motor predictors, including extreme residuals (left), and with extreme residuals removed (right).

Cognitive Predictors of Tapping Force for the Dual Task Cost

Figure E5. Q-Q Plot of Residuals of the model with cognitive predictors of tapping force for the dual task cost.

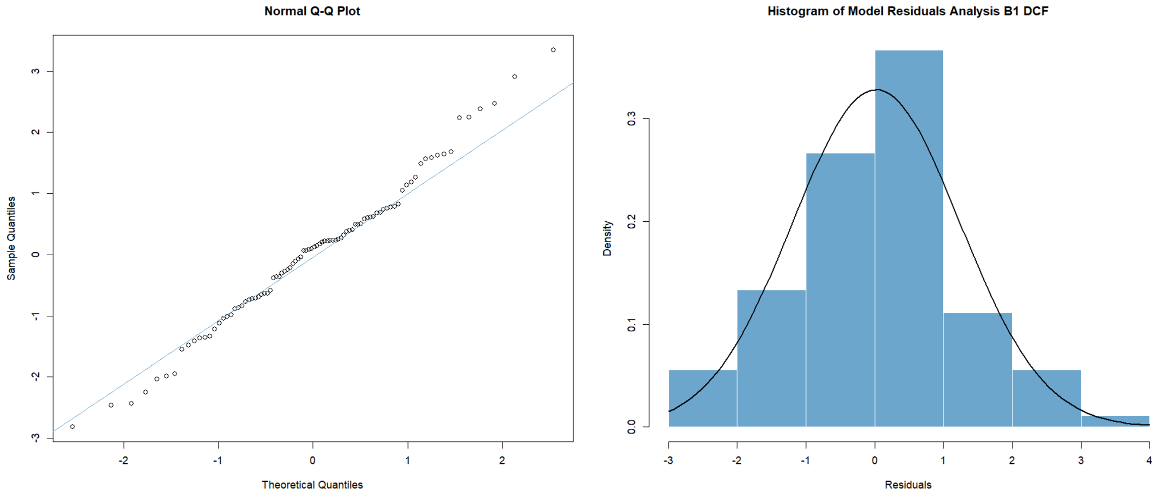

Note. Residuals from the model of Dual Task Cost tapping force with cognitive predictors showing a QQ-plot (left), and a histogram of residuals (right).

Cognitive Predictors of Tapping Consistency for the Dual Task Cost

Figure E6. Q-Q Plot of Residuals of the model with cognitive predictors of tapping consistency for the dual task cost.

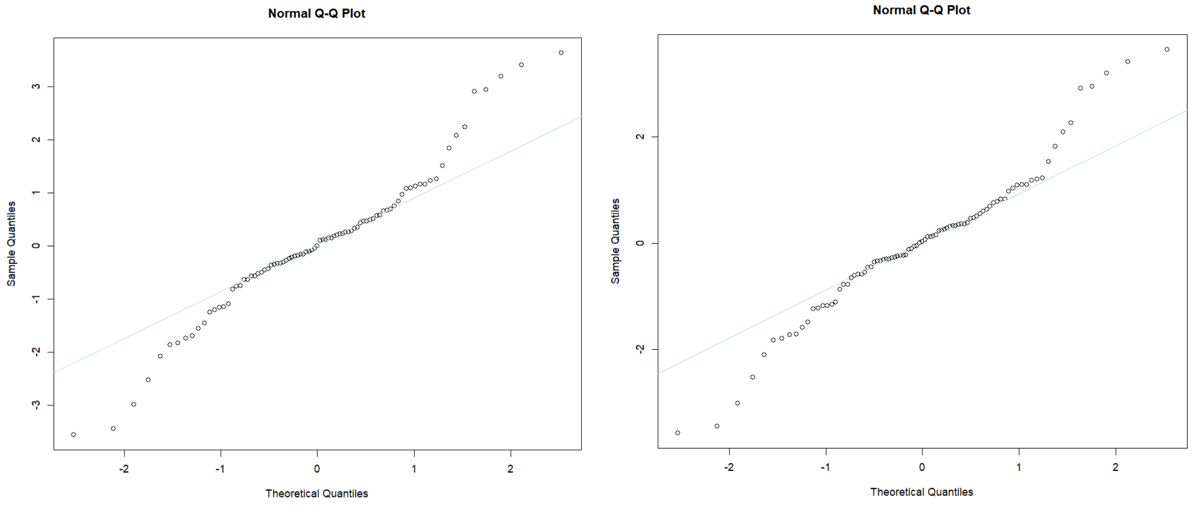

Note. QQ-plot of residuals from the model of Dual Task Cost tapping consistency with cognitive predictors, including extreme residuals (left), and with extreme residuals removed (right).

Motor Predictors of Tapping Force for the Dual Task Cost

Figure E7. Q-Q Plot of Residuals of the model with motor predictors of tapping force for the dual task cost.

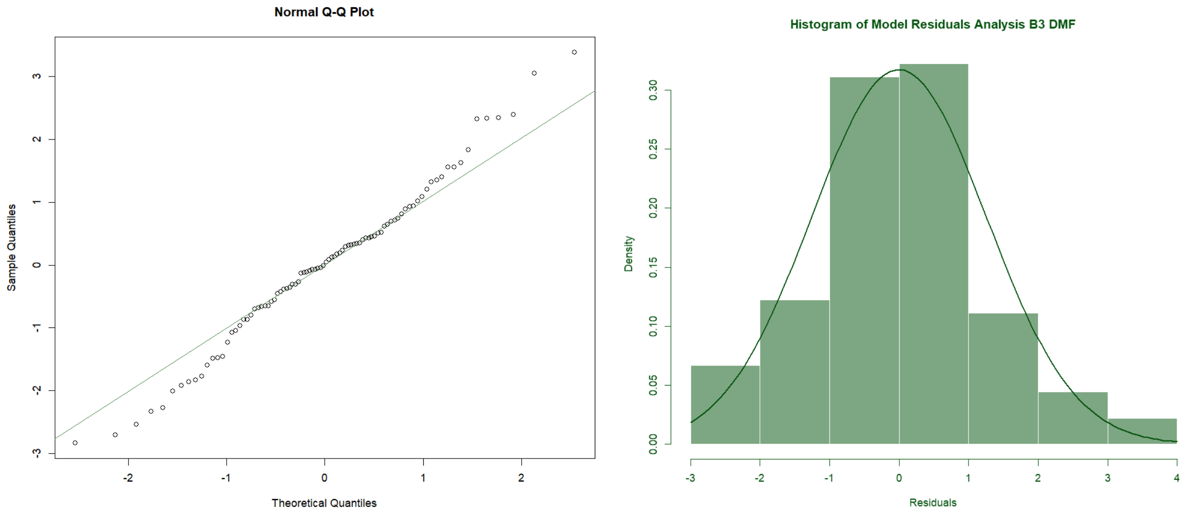

Note. Residuals from the model of Dual Task Cost tapping force with motor predictors showing a QQ-plot (left), and a histogram of residuals (right).

Motor Predictors of Tapping Consistency for the Dual Task Cost

Figure E15. . Q-Q Plot of Residuals of the model with Motor Predictors of tapping consistency for the dual task cost.

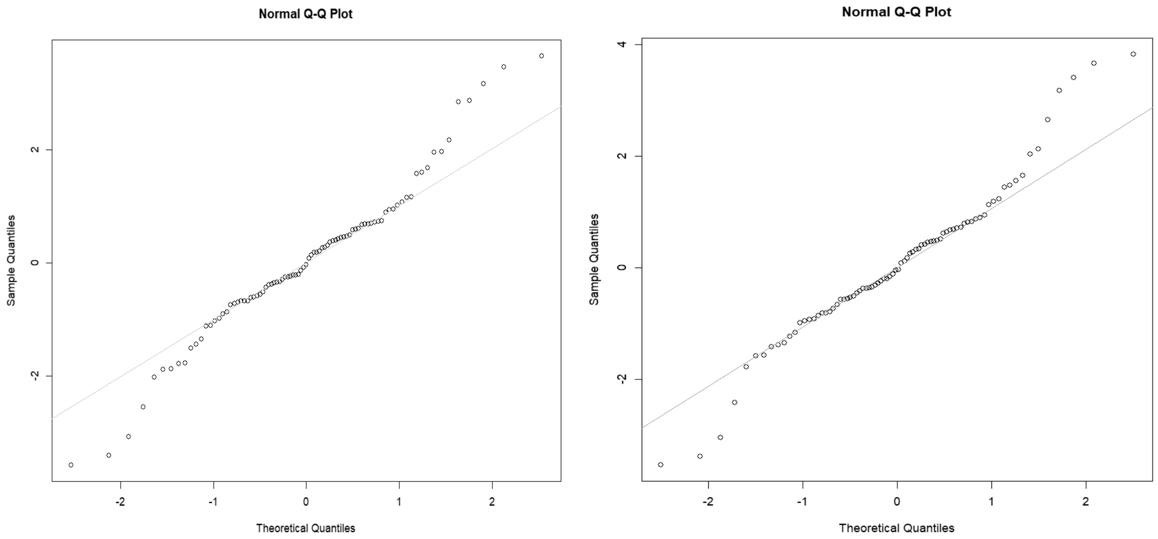

Note. QQ-plot of residuals from the model of Dual Task Cost of tapping consistency with motor predictors, including extreme residuals (left), and with extreme residuals removed (right).
