# Supplementary material for: Cognitive and motor abilities predict auditory-cued finger tapping in a dual task
Source: Front Neurosci. 2025 May 21;19:1553548. doi: 10.3389/fnins.2025.1553548 (PMC12133802; doi:10.3389/fnins.2025.1553548)
Supplement: Supplementary file 6 [file Data_Sheet_6.pdf]

Supplementary Material F

GAMs including Musical Questionnaires

Figure F1. Summary of cognitive, motor, musical predictors of tapping force and consistency for ST, DT, and DTC.

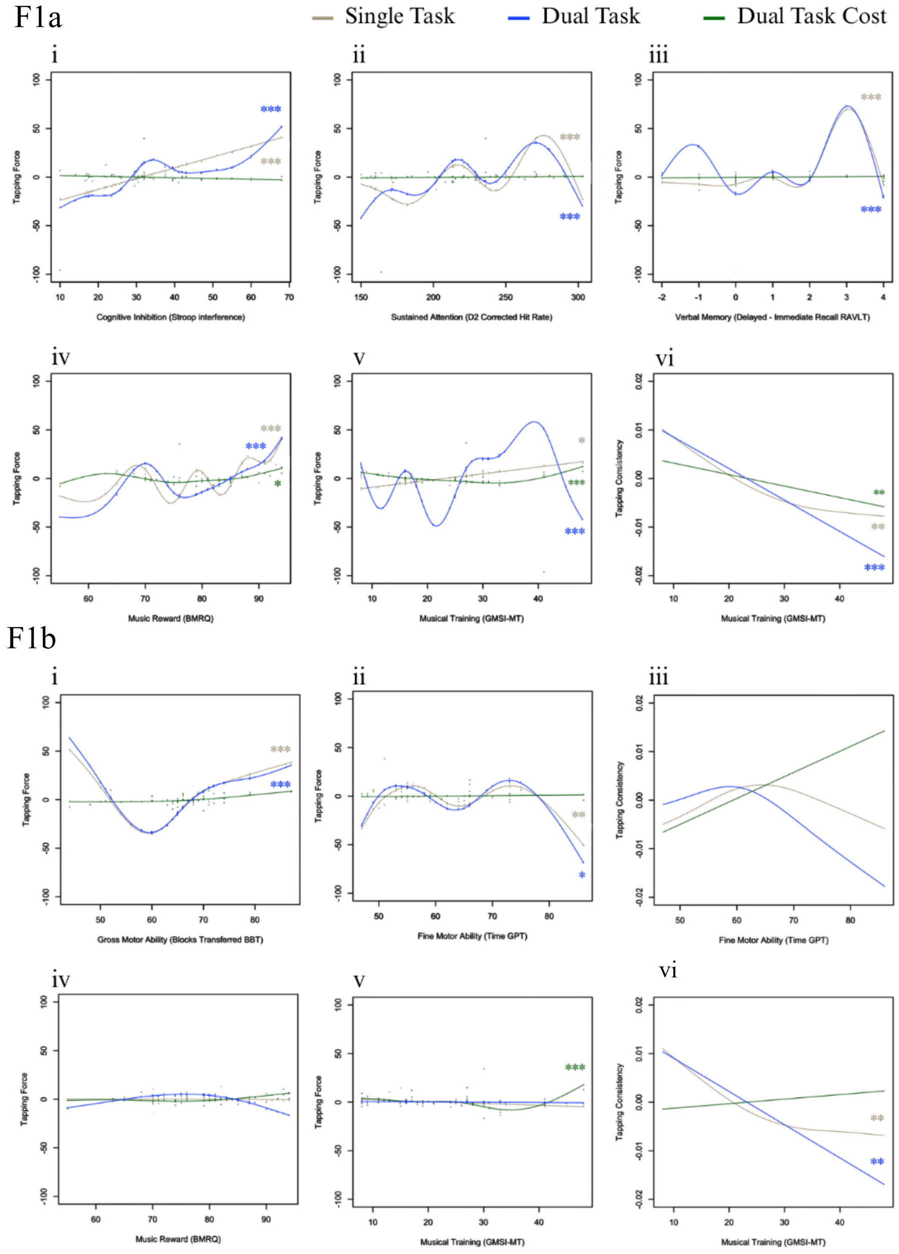

Note. Panel (a – top) shows the models with cognitive and musical predictors of tapping performance across ST, DT, and DTC. Graph i) show Stroop interference on the x-axis (with greater numbers indicating greater interference, i.e., worse performance), and tapping force on the y-axis. Graphs (a) ii, and iii show outcomes of D2, where higher scores indicate better performance, and RAVLT, where higher scores indicate discrepancy between immediate and delayed memory recall. Graph (a) iv and v show musical predictors of tapping force, and vi shows musical training as predictor of tapping consistency.
Panel (b – bottom) shows the models with motor and musical predictors of tapping performance across ST, DT, and DTC. Graphs i) shows BBT (higher score better performance, and ii) GPT (where higher scores indicate worse performance), and tapping force on the y-axis, whereas iii) shows tapping consistency on the y-axis. Graph iv, and v show musical predictors of tapping force in the model with motor predictors, whereas vi, shows musical training as a predictor of tapping consistency.
Significant Bonferroni-corrected results are indicated by (*) for *p*-values < .05, (**) for *p*-values < .01, and (***) for *p*-values <0.001.

Models of the Single Task Performance

Table F1. *GAMs Results of Cognitive and Musical Predictors of Tapping Force in the Single Task.*

Smoothing terms	*Edf*	*df*	χ^2	*p*	*Bonferroni α*
s(RAVLT)	5.73	5.94	57.01	< .001***	< .001***
s(Stroop)	1.00	1.00	17.04	< .001***	< .001***
s(TMT B-A)	1.81	2.15	1.78	.428	.856
s(D2)	6.82	7.78	54.63	< .001***	< .001***
s(GMSI MT)	1.00	1.00	6.34	.012*	.024*
s(BMRQ)	7.68	8.34	33.12	< .001***	< .001***
s(PPT)	0.85	1.00	5.73	.006**	.012*

Parametric coefficients	Estimate	*SE*	*z*	*p*	*Bonferroni α*
(Intercept)	74.11	7.07	10.48	< .001***	< .001***
Auditory Cue	0.64	3.60	0.18	.858	1.00
R^2 (adj.) .686		Deviance explained		77.7%	

Note. Signif. codes: '***' 0.001 '**' 0.01 '*' 0.05. RAVLT = Rey Auditory Verbal Learning Test calculated as 5th Immediate Trial Recall – Delayed Recalled Items; Stroop = calculated as Incongruent – Congruent Trials Time in seconds; TMT = Trail Making Test calculated as Switching – Counting Time (B-A) in seconds; D2 calculated as corrected hit rate (correct hits – false positives); GMSI MT= Musical Training subscale of the Gold Music Sophistication Index; BMRQ = total score on the Barcelona Music Rating Questionnaire; PPT = Participants.

Formula:
FORCE_ST ~ s(TMT_B_A_Time, k = -1) + s(STROOP_CWI, k = -1) +
s(D2_CHR, k = -1) + s(RAVLT_T5_DL, k = 7) + s(GMSI_MT, k = -1) +
s(BMRQ, k = -1) + Condition + s(PPT, bs = "re")

Figure F2. *Partial Effect Plots GAM Single Task Cognition and Tapping Force including Musical Questionnaires.*

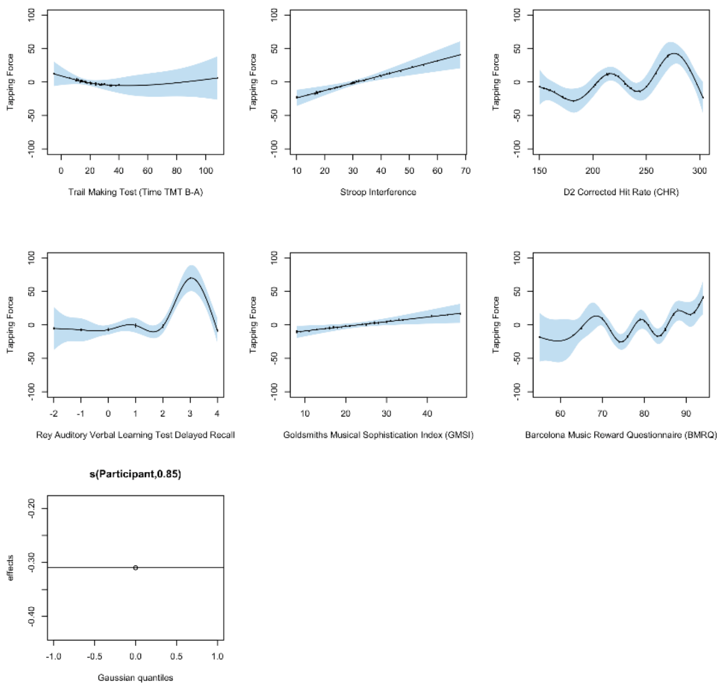

Note. Graphs visualize results with residual outliers included. The solid line represents the fitted relationship, and the shaded area represents the 95% confidence interval of the estimated smooth effect.

Table F2. *GAMs Results of Cognitive and Musical Predictors of Tapping Consistency in the Single Task.*

Smoothing terms	*Edf*	*df*	χ^2	*p*	*Bonferroni α*
s(RAVLT)	1.00	1.00	0.65	.419	.838
s(Stroop)	1.93	2.41	2.88	.295	.590
s(TMT B-A)	1.00	1.00	0.01	.925	1
s(D2)	1.00	1.00	0.26	.613	1
s(GMSI MT)	1.83	2.27	12.94	.002**	.004**
s(BMRQ)	2.27	2.83	3.36	.219	.438
s(PPT)	0.00	1.00	0.00	.819	1

Parametric coefficients	Estimate	*SE*	*z*	*p*	*Bonferroni α*
(Intercept)	4.39×10^{-02}	4.34×10^{-03}	10.10	< .001***	< .001***
Auditory Cue	1.20×10^{-03}	2.74×10^{-03}	0.44	.66	.132
R^2 (adj.)	-.115	Deviance explained	13.6%		

Note. Results with residual outliers included. Signif. codes: '***' 0.001 '**' 0.01 '*' 0.05. RAVLT = Rey Auditory Verbal Learning Test calculated as 5th Immediate Trial Recall – Delayed Recalled Items; Stroop = calculated as Incongruent – Congruent Trials Time in seconds; TMT = Trail Making Test calculated as Switching – Counting Time (B-A) in seconds; D2 calculated as corrected hit rate (correct hits – false positives); GMSI MT= Musical Training subscale of the Gold Music Sophistication Index; BMRQ = total score on the Barcelona Music Rating Questionnaire; PPT = Participants.

Formula:
CV_ST ~ s(TMT_B_A_Time, k = -1) + s(STROOP_CWI, k = -1) + s(D2_CHR, k = -1) + s(RAVLT_T5_DL, k = 7) + s(GMSI_MT, k = -1) + s(BMRQ, k = -1) + Condition + s(PPT, bs = "re")

Figure F3. *Partial Effect Plots GAM Single Task Cognition and Tapping Consistency including Musical Questionnaires.*

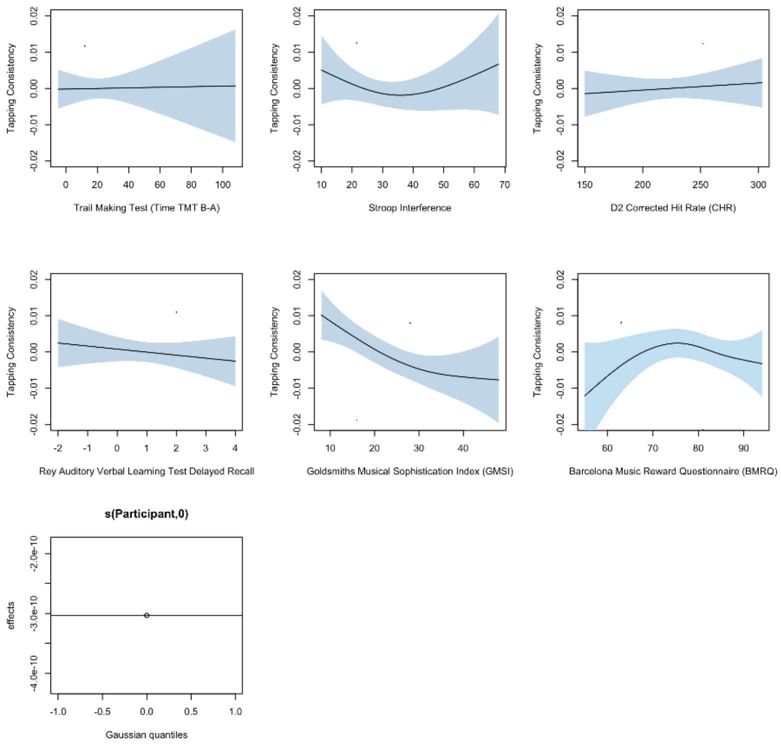

Note. Graphs visualize results with residual outliers included. The solid line represents the fitted relationship, and the shaded area represents the 95% confidence interval of the estimated smooth effect.

Table F3. *Results Summary GAM Motor and Musical Predictors and Tapping Force in the Single Task*

Smoothing terms		*Edf*	*df*	χ^2	*p*	*Bonferroni α*
s(GPT)		5.00	5.95	20.11	.003**	.006**
s(BBT)		4.78	5.72	61.42	< .001***	< .001***
s(GMSI MT)		1.00	1.00	0.45	.505	1
s(BMRQ)		1.00	1.00	0.00	.956	1
s(PPT)		0.78	1.00	3.55	.029*	.058
Parametric coefficients	Estimate	*SE*	*z*		*p*	*Bonferroni α*
(Intercept)	70.74	8.12	8.71		< .001***	< .001***
Auditory Cue	0.64	4.62	0.14		.889	1
R^2 (adj.)	.483		Deviance explained		56.2%	

Note. Signif. codes: '***' 0.001 '**' 0.01 '*' 0.05. GPT = Grooved Pegboard Task calculated as time to complete in seconds; BBT= Box and Blocks Test calculated as total count of transferred blocks; GMSI MT= Musical Training subscale of the Gold Music Sophistication Index; BMRQ = total score on the Barcelona Music Rating Questionnaire; PPT = Participants.

Formula:
FORCE_ST ~ s(GPT_TIME_DH, k = -1) + s(BBT_DH_COUNT, k = -1) +
s(GMSI_MT, k = -1) + s(BMRQ, k = -1) + Condition + s(PPT,
bs = "re")

Figure F4. *Partial Effect Plots GAM Single Task Motor Ability and Tapping Force including Musical Questionnaires.*

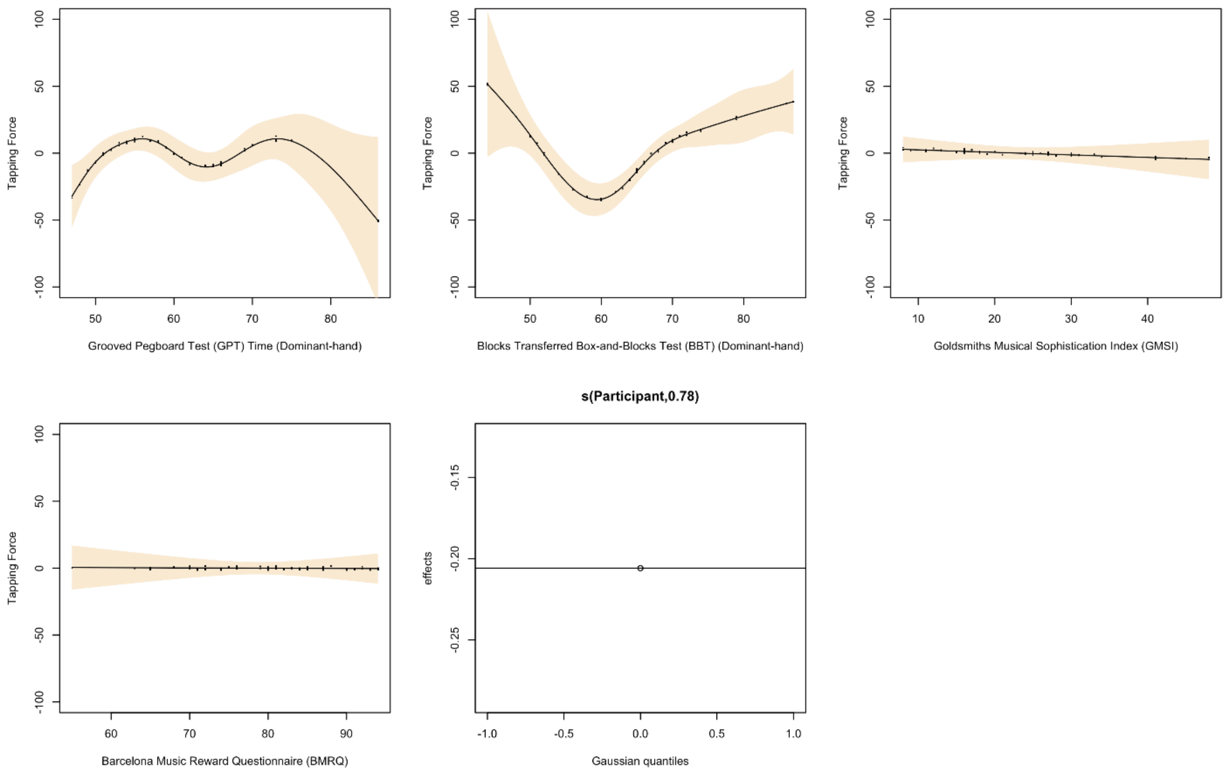

Note. Graphs visualize results with residual outliers included. The solid line represents the fitted relationship, and the shaded area represents the 95% confidence interval of the estimated smooth effect.

Table F4. *Results Summary GAM Motor and Musical Predictors and Tapping Consistency in the Single Task*

Smoothing terms		*Edf*	*df*	χ^2	*p*	*Bonferroni α*
s(GPT)		2.11	2.66	3.87	.237	.474
s(BBT)		1.00	1.00	0.71	.399	.798
s(GMSI MT)		2.02	2.50	13.69	.002**	.004**
s(BMRQ)		2.13	2.67	1.54	.474	.948
s(PPT)		1.59×10^{-04}	1.00	0.00	.307	.614
Parametric coefficients	Estimate	*SE*	*z*		*p*	*Bonferroni α*
(Intercept)	4.56×10^{-02}	4.34×10^{-03}	10.49		< .001***	< .001***
Auditory Cue	1.00×10^{-04}	2.74×10^{-03}	0.04		.971	1
R^2 (adj.)	-.098	Deviance explained			14.7%	

Note. Signif. codes: '***' 0.001 '**' 0.01 '*' 0.05. GPT = Grooved Pegboard Task calculated as time to complete in seconds; BBT= Box and Blocks Test calculated as total count of transferred blocks; GMSI MT= Musical Training subscale of the Gold Music Sophistication Index; BMRQ = total score on the Barcelona Music Rating Questionnaire; PPT = Participants.

Formula:
CV_ST ~ s(GPT_TIME_DH, k = -1) + s(BBT_DH_COUNT, k = -1) + s(GMSI_MT, k = -1) + s(BMRQ, k = -1) + Condition + s(PPT, bs = "re")

Figure F5. *Partial Effect Plots GAM Single Task Motor Ability and Tapping Consistency including Musical Questionnaires.*

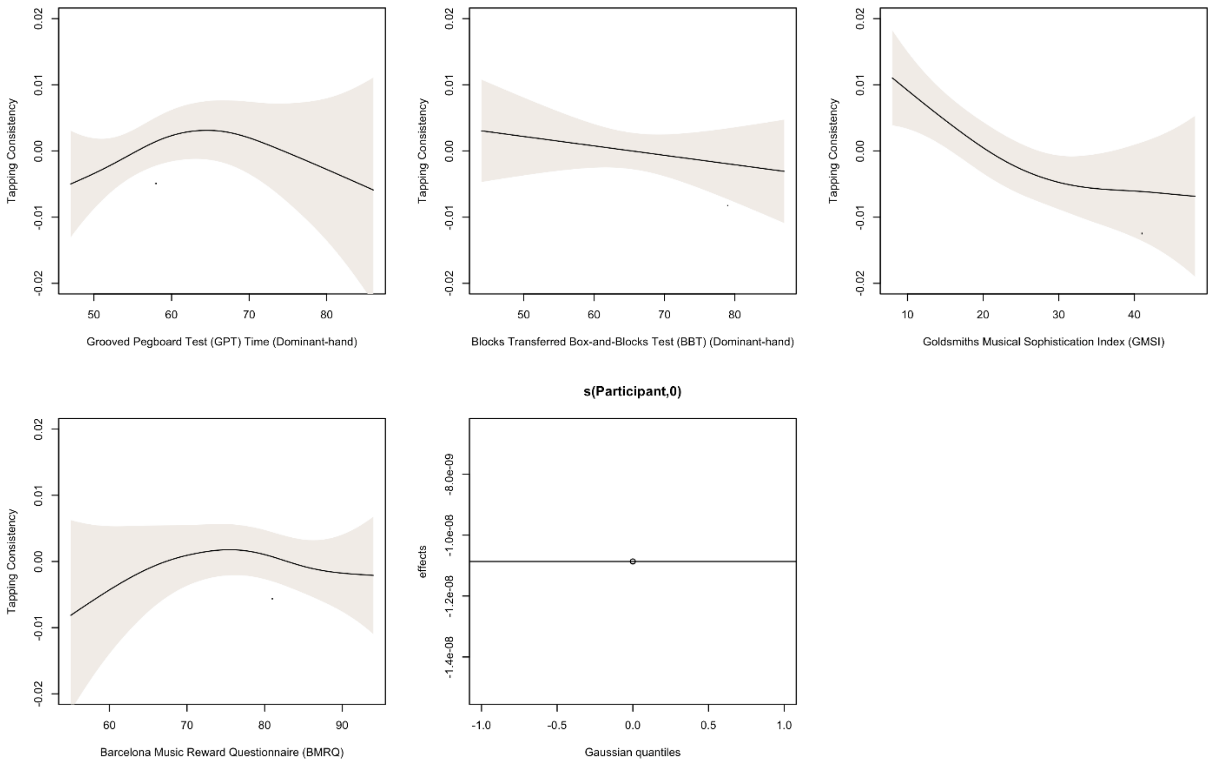

Note. Graphs visualize results with residual outliers included. The solid line represents the fitted relationship, and the shaded area represents the 95% confidence interval of the estimated smooth effect.

Models of the Dual Task Cost Performance

Table F5. *Results Summary GAM Dual Task Cost: Cognitive and Musical Predictors of Tapping Force.*

Smoothing terms		*Edf*	*df*	χ^2	*p*	*Bonferroni α*
s(RAVLT)		1.00	1.00	0.12	.726	1
s(Stroop)		1.00	1.00	0.63	.428	.856
s(TMT B-A)		1.00	1.00	0.57	.449	.898
s(D2)		1.00	1.00	0.13	.715	1
s(GMSI MT)		3.32	4.10	21.32	< .001 ***	< .001 ***
s(BMRQ)		4.29	5.25	17.50	.005 **	.010*
s(PPT)		0.75	1.00	3.01	.038*	.076
Parametric coefficients	Estimate	*SE*	*z*		*p*	*Bonferroni α*
(Intercept)	-1.90	3.04	-0.63		.532	1
Auditory Cue	2.02	1.71	1.18		.238	.476
R^2 (adj.)	.265		Deviance explained		25.8%	

Note. Signif. codes: '***' 0.001 '**' 0.01 '*' 0.05. Results with residual outliers included. RAVLT = Rey Auditory Verbal Learning Test calculated as 5th Immediate Trial Recall – Delayed Recalled Items; Stroop = calculated as Incongruent – Congruent Trials Time in seconds; TMT = Trail Making Test calculated as Switching – Counting Time (B-A) in seconds; D2 calculated as corrected hit rate (correct hits – false positives); GMSI MT= Musical Training subscale of the Gold Music Sophistication Index; BMRQ = total score on the Barcelona Music Rating Questionnaire; PPT = Participants.

Formula:
FORCE_DTC ~ s(TMT_B_A_Time, k = -1) + s(STROOP_CWI, k = -1) + s(D2_CHR, k = -1) + s(RAVLT_T5_DL, k = 7) + s(GMSI_MT, k = -1) + s(BMRQ, k = -1) + Condition + s(PPT, bs = "re")

Figure F6. *Partial Effect Plots GAM Dual Task Cost: Cognition and Tapping Force including Musical Questionnaires.*

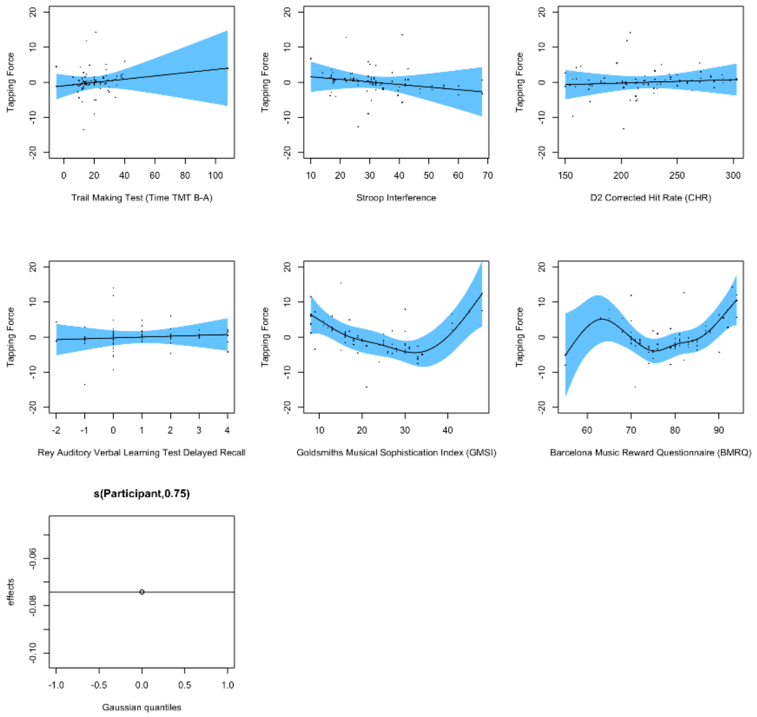

Note. Graphs visualize results with residual outliers included. The solid line represents the fitted relationship, and the shaded area represents the 95% confidence interval of the estimated smooth effect.

Table F6. *Results Summary GAM Dual Task Cost: Cognitive and Musical Predictors and Tapping Consistency*

Smoothing terms	*Edf*	*df*	χ^2	*p*	*Bonferroni α*
s(RAVLT)	1.00	1.00	0.16	.690	1
s(Stroop)	1.00	1.00	1.65	.200	.400
s(TMT B-A)	3.02	3.64	6.73	.106	.212
s(D2)	1.00	1.00	0.73	.392	.784
s(GMSI MT)	1.00	1.00	1.04	.308	.616
s(BMRQ)	1.00	1.00	0.12	.732	1
s(PPT)	3.35×10^{-06}	1.00	0.00	.408	.816

Parametric coefficients	Estimate	*SE*	*z*	*p*	*Bonferroni α*
(Intercept)	-1.1×10^{-02}	5.9×10^{-03}	-1.89	.059	.118
Auditory Cue	1.5×10^{-03}	3.7×10^{-03}	0.40	.693	1
R^2 (adj.)	-.031	Deviance explained		7.26%	

Note. Results with residual outliers included. Signif. codes: '***' 0.001 '**' 0.01 '*' 0.05. RAVLT = Rey Auditory Verbal Learning Test calculated as 5th Immediate Trial Recall – Delayed Recalled Items; Stroop = calculated as Incongruent – Congruent Trials Time in seconds; TMT = Trail Making Test calculated as Switching – Counting Time (B-A) in seconds; D2 calculated as corrected hit rate (correct hits – false positives); GMSI MT= Musical Training subscale of the Gold Music Sophistication Index; BMRQ = total score on the Barcelona Music Rating Questionnaire; PPT = Participants.

Formula:
CV_DTC ~ s(TMT_B_A_Time, k = -1) + s(STROOP_CWI, k = -1) + s(D2_CHR, k = -1) + s(RAVLT_T5_DL, k = 7) + s(GMSI_MT, k = -1) + s(BMRQ, k = -1) + Condition + s(PPT, bs = "re")

Figure F7. *Partial Effect Plots GAM Dual Task Cost: Cognition and Tapping Consistency including Musical Questionnaires.*

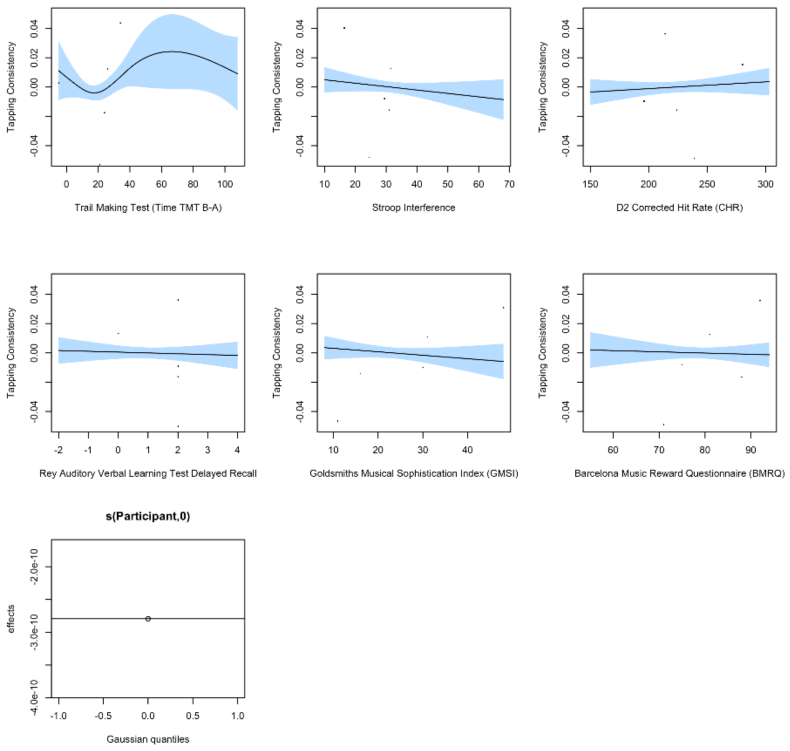

Note. Graphs visualize results with residual outliers included. The solid line represents the fitted relationship, and the shaded area represents the 95% confidence interval of the estimated smooth effect.

Table F7. *Results Summary GAM Dual Task Cost: Motor and Musical Predictors and Tapping Force.*

Smoothing terms		*Edf*	*df*	χ^2	*p*	*Bonferroni α*
s(GPT)		1.00	1.00	0.16	.693	1
s(BBT)		1.90	2.36	7.35	.039*	.078
s(GMSI MT)		4.63	5.64	27.86	< .001***	< .001***
s(BMRQ)		2.33	2.91	6.90	.084	.168
s(PPT)		0.03	1.00	0.05	.235	.470
Parametric coefficients	Estimate	*SE*	*z*		*p*	*Bonferroni α*
(Intercept)	-4.39	2.64	-1.66		.096	.192
Auditory Cue	2.05	1.66	1.23		.217	.434
R^2 (adj.)	.290	Deviance explained			26.1%	

Note. Signif. codes: ‘***’ 0.001 ‘**’ 0.01 ‘*’ 0.05. GPT = Grooved Pegboard Task calculated as time to complete in seconds; BBT= Box and Blocks Test calculated as total count of transferred blocks; GMSI MT= Musical Training subscale of the Gold Music Sophistication Index; BMRQ = total score on the Barcelona Music Rating Questionnaire; PPT = Participants.

Formula:
FORCE_DTC ~ s(GPT_TIME_DH, k = -1) + s(BBT_DH_COUNT, k = -1) +
s(GMSI_MT, k = -1) + s(BMRQ, k = -1) + Condition + s(PPT,
bs = "re")

Figure B7. *Partial Effect Plots GAM Dual Task Cost: Motor Ability and Tapping Force including Musical Questionnaires.*

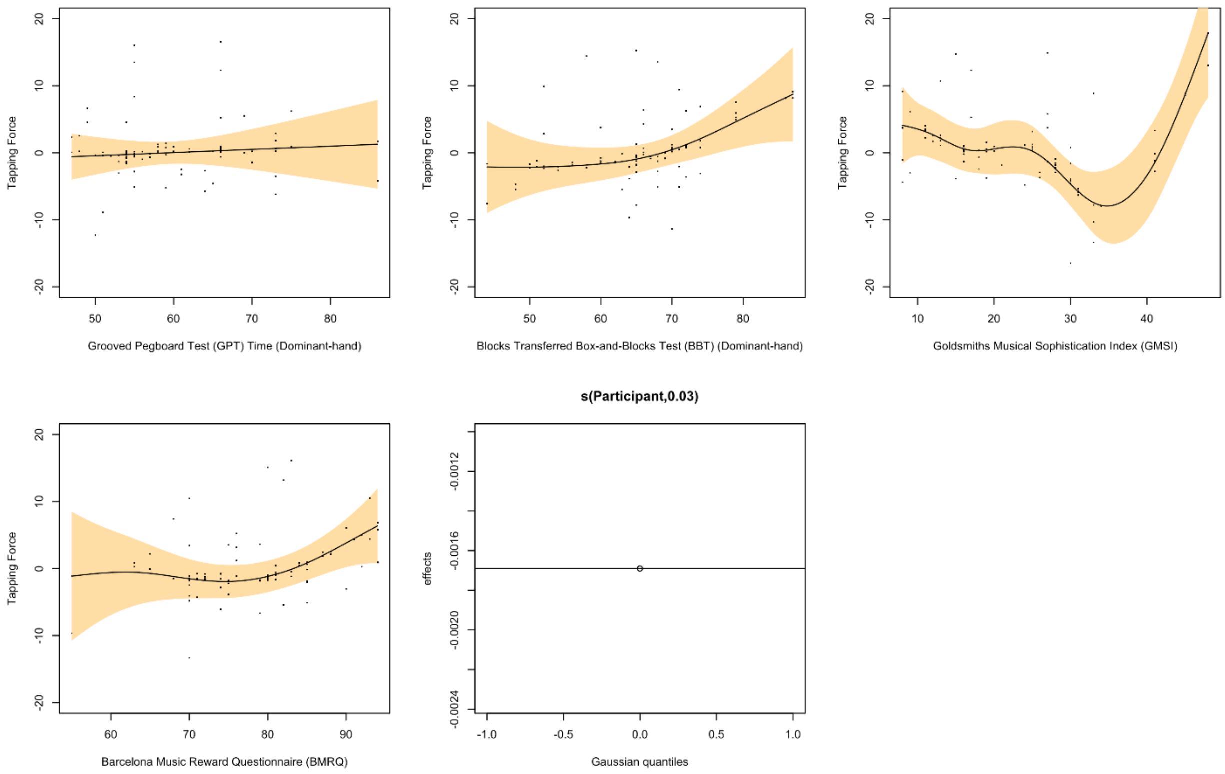

Note. Graphs visualize results with residual outliers included. The solid line represents the fitted relationship, and the shaded area represents the 95% confidence interval of the estimated smooth effect.

Table F8. *Results Summary GAM Dual Task Cost: Motor and Musical Predictors and Tapping Consistency.*

Smoothing terms		*Edf*	*df*	χ^2	*p*	*Bonferroni α*
s(GPT)		1.00	1.00	4.21	.040*	.080
s(BBT)		1.00	1.00	2.24	.134	.268
s(GMSI MT)		1.00	1.00	0.194	.659	1
s(BMRQ)		1.00	1.00	2.054	.152	.304
s(PPT)		2.13×10^{-06}	1.00	0.00	.948	1
Parametric coefficients	Estimate	*SE*	*z*		*p*	*Bonferroni α*
(Intercept)	-0.01	0.01	-1.73		.084	.168
Auditory Cue	7.34×10^{-04}	3.76×10^{-03}	0.20		.845	1
R^2 (adj.)	-.046	Deviance explained		3.95%		

Note. Signif. codes: '***' 0.001 '**' 0.01 '*' 0.05. GPT = Grooved Pegboard Task calculated as time to complete in seconds; BBT= Box and Blocks Test calculated as total count of transferred blocks; GMSI MT= Musical Training subscale of the Gold Music Sophistication Index; BMRQ = total score on the Barcelona Music Rating Questionnaire; PPT = Participants.

```
Formula:
CV_DTC ~ s(GPT_TIME_DH, k = -1) + s(BBT_DH_COUNT, k = -1) + s(GMSI_MT,
    k = -1) + s(BMRQ, k = -1) + Condition + s(PPT, bs = "re")
```

Figure F9. *Partial Effect Plots GAM Dual Task Cost: Motor Ability and Tapping Consistency including Musical Questionnaires.*

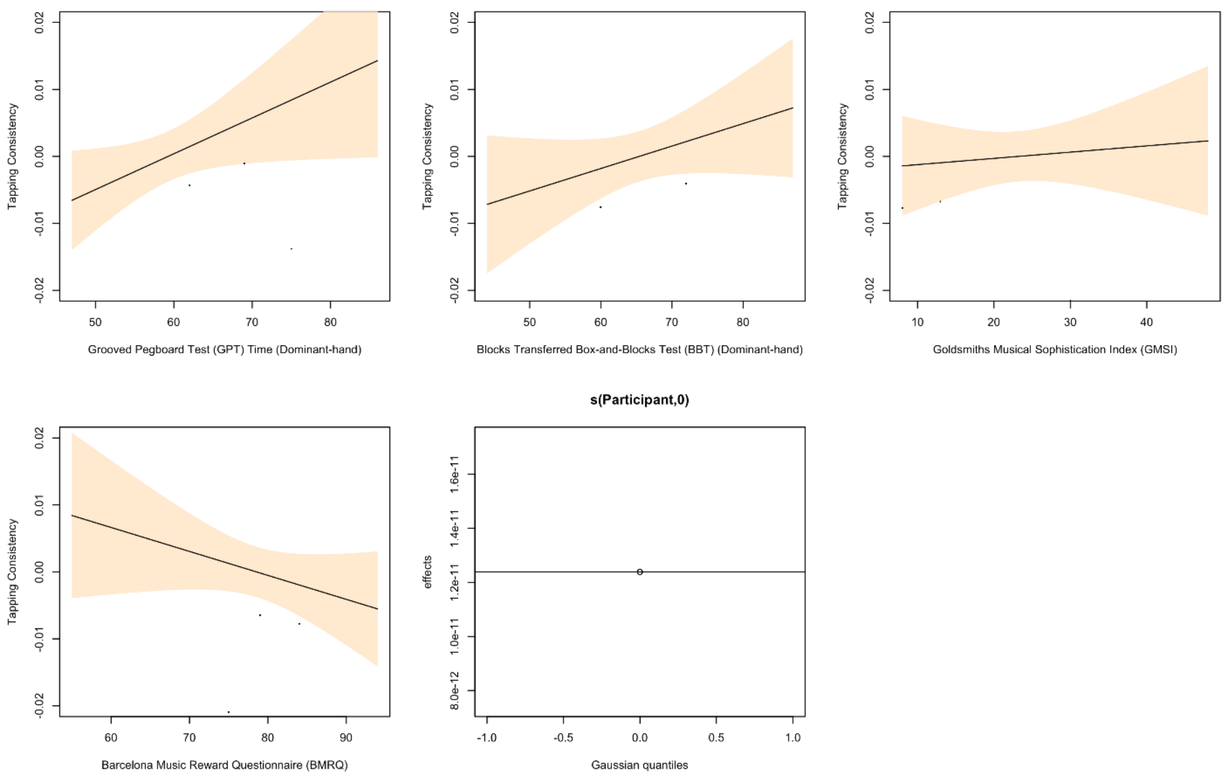

Note. Graphs visualize results with residual outliers included. The solid line represents the fitted relationship, and the shaded area represents the 95% confidence interval of the estimated smooth effect.

Models of the Dual Task Performance

Table F9. *Results Summary GAM Cognitive and Musical Predictors and Tapping Force in the Dual Task.*

Smoothing terms		*Edf*	*df*	χ^2	*p*	*Bonferroni α*
s(RAVLT)		5.78	5.92	94.74	< .001***	< .001***
s(Stroop)		5.45	6.31	33.20	< .001***	< .001***
s(TMT B-A)		1.00	1.00	1.69	.194	.388
s(D2)		6.89	7.70	40.86	< .001***	< .001***
s(GMSI MT)		8.05	8.56	77.17	< .001***	< .001***
s(BMRQ)		6.11	6.81	30.38	< .001***	< .001***
s(PPT)		0.90	1.00	9.11	< .001***	< .001***
Parametric coefficients	Estimate	*SE*	*z*		*p*	*Bonferroni α*
(Intercept)	80.42	6.00	13.40		< .001***	< .001***
Auditory Cue	-0.89	2.75	-0.32		.747	1
R^2 (adj.)	.826		Deviance explained		89.5%	

Note. Results with residual outliers included. Signif. codes: '***' 0.001 '**' 0.01 '*' 0.05. RAVLT = Rey Auditory Verbal Learning Test calculated as 5th Immediate Trial Recall – Delayed Recalled Items; Stroop = calculated as Incongruent – Congruent Trials Time in seconds; TMT = Trail Making Test calculated as Switching – Counting Time (B-A) in seconds; D2 calculated as corrected hit rate (correct hits – false positives); GMSI MT= Musical Training subscale of the Gold Music Sophistication Index; BMRQ = total score on the Barcelona Music Rating Questionnaire; PPT = Participants.

FORCE_DT ~ s(TMT_B_A_Time, k = -1) + s(STROOP_CWI, k = -1) +
s(D2_CHR, k = -1) + s(RAVLT_T5_DL, k = 7) + s(GMSI_MT, k = -1) +
s(BMRQ, k = -1) + Condition + s(PPT, bs = "re")

Figure F10. *Partial Effect Plots GAM Cognitive Predictors of Tapping Force in the Dual Task including Musical Questionnaires.*

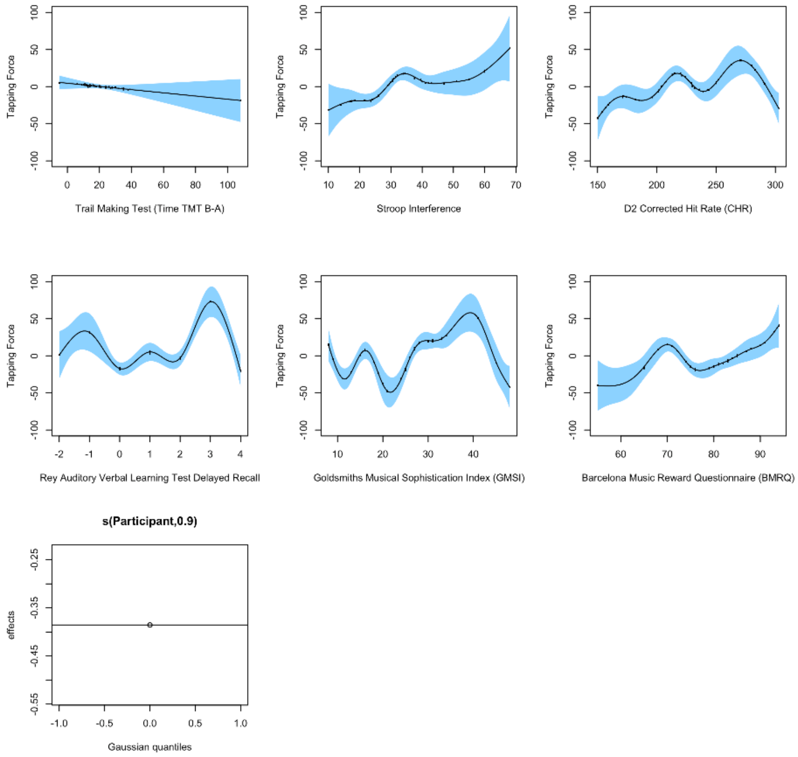

Note. Graphs visualize results when residual outliers are included. The solid line represents the fitted relationship, and the shaded area represents the 95% confidence interval of the estimated smooth effect.

Table F10. *Results Summary GAM Cognitive and Musical Predictors and Tapping Consistency in the Dual Task.*

Smoothing terms		*Edf*	*df*	χ^2	*p*	*Bonferroni α*
s(RAVLT)		1.00	1.00	0.774	.379	.758
s(Stroop)		1.88	2.36	3.53	.209	.418
s(TMT B-A)		1.36	1.61	1.08	.374	.748
s(D2)		1.00	1.00	1.52	.218	.436
s(GMSI MT)		1.00	1.00	9.731	.002**	.004**
s(BMRQ)		1.00	1.00	1.145	.285	.570
s(PPT)		5.01×10^{-05}	1.00	0.00	.479	.958
Parametric coefficients	Estimate	*SE*	*z*		*p*	*Bonferroni α*
(Intercept)	0.06	0.01	9.93		< .001***	< .001***
Auditory Cue	-1.12×10^{-03}	0.00	-0.31		.754	1
R^2 (adj.)	-.075	Deviance explained			11.3%	

Note. Results with residual outliers included. Signif. codes: '***' 0.001 '**' 0.01 '*' 0.05. RAVLT = Rey Auditory Verbal Learning Test calculated as 5th Immediate Trial Recall – Delayed Recalled Items; Stroop = calculated as Incongruent – Congruent Trials Time in seconds; TMT = Trail Making Test calculated as Switching – Counting Time (B-A) in seconds; D2 calculated as corrected hit rate (correct hits – false positives); GMSI MT= Musical Training subscale of the Gold Music Sophistication Index; BMRQ = total score on the Barcelona Music Rating Questionnaire; PPT = Participants.

Formula:
CV_DT ~ s(TMT_B_A_Time, k = -1) + s(STROOP_CWI, k = -1) + s(D2_CHR, k = -1) + s(RAVLT_T5_DL, k = 7) + s(GMSI_MT, k = -1) + s(BMRQ, k = -1) + Condition + s(PPT, bs = "re")

Figure F11. *Partial Effect Plots GAM Cognitive Predictors of Tapping Consistency in the Dual Task including Musical Questionnaires.*

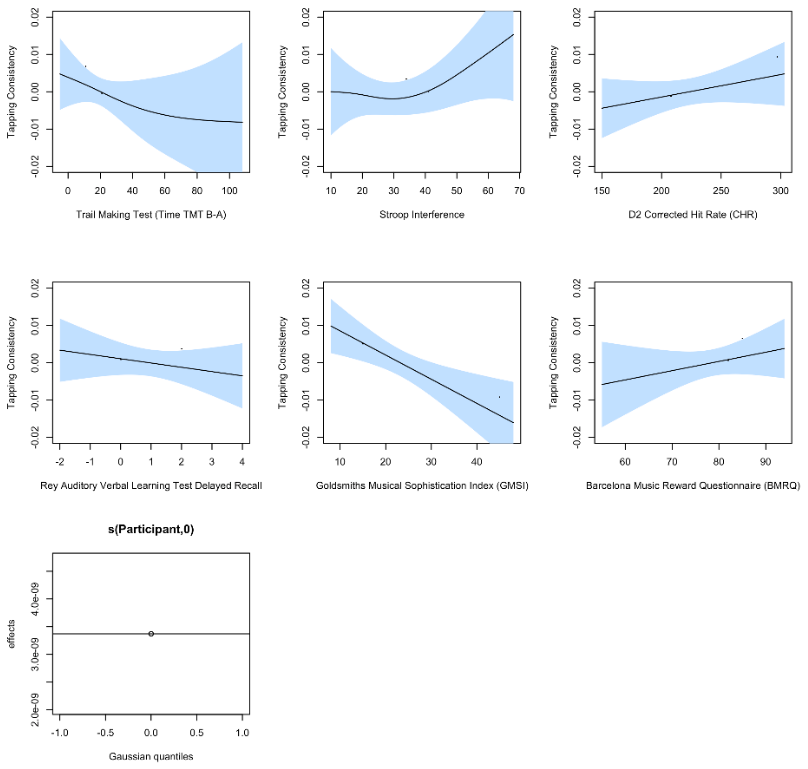

Note. Graphs visualize results when residual outliers are included. The solid line represents the fitted relationship, and the shaded area represents the 95% confidence interval of the estimated smooth effect.

Table F11. *Results Summary GAM Motor and Musical Predictors and Tapping Force in the Dual Task.*

Smoothing terms		*Edf*	*df*	χ^2	*p*	*Bonferroni α*
s(GPT)		5.23	6.17	19.90	.004**	.008**
s(BBT)		4.61	5.50	46.26	< .001***	< .001***
s(GMSI MT)		1.00	1.00	0.01	.926	1
s(BMRQ)		2.24	2.77	4.62	.173	.346
s(PPT)		0.37	1.00	0.59	.192	.384
Parametric coefficients	Estimate	*SE*	*z*		*p*	*Bonferroni α*
(Intercept)	70.07	8.50	8.24		< .001***	< .001***
Auditory Cue	-0.89	5.08	-0.18		0.861	1
R^2 (adj.)	-.407	Deviance explained			50.4%	

Note. Signif. codes: '***' 0.001 '**' 0.01 '*' 0.05. GPT = Grooved Pegboard Task calculated as time to complete in seconds; BBT= Box and Blocks Test calculated as total count of transferred blocks; GMSI MT= Musical Training subscale of the Gold Music Sophistication Index; BMRQ = total score on the Barcelona Music Rating Questionnaire; PPT = Participants.

Formula:
FORCE_DT ~ s(GPT_TIME_DH, k = -1) + s(BBT_DH_COUNT, k = -1) +
s(GMSI_MT, k = -1) + s(BMRQ, k = -1) + Condition + s(PPT,
bs = "re")

Figure F12. *Partial Effect Plots GAM Motor Predictors and Tapping Force in the Dual Task including Musical Questionnaires.*

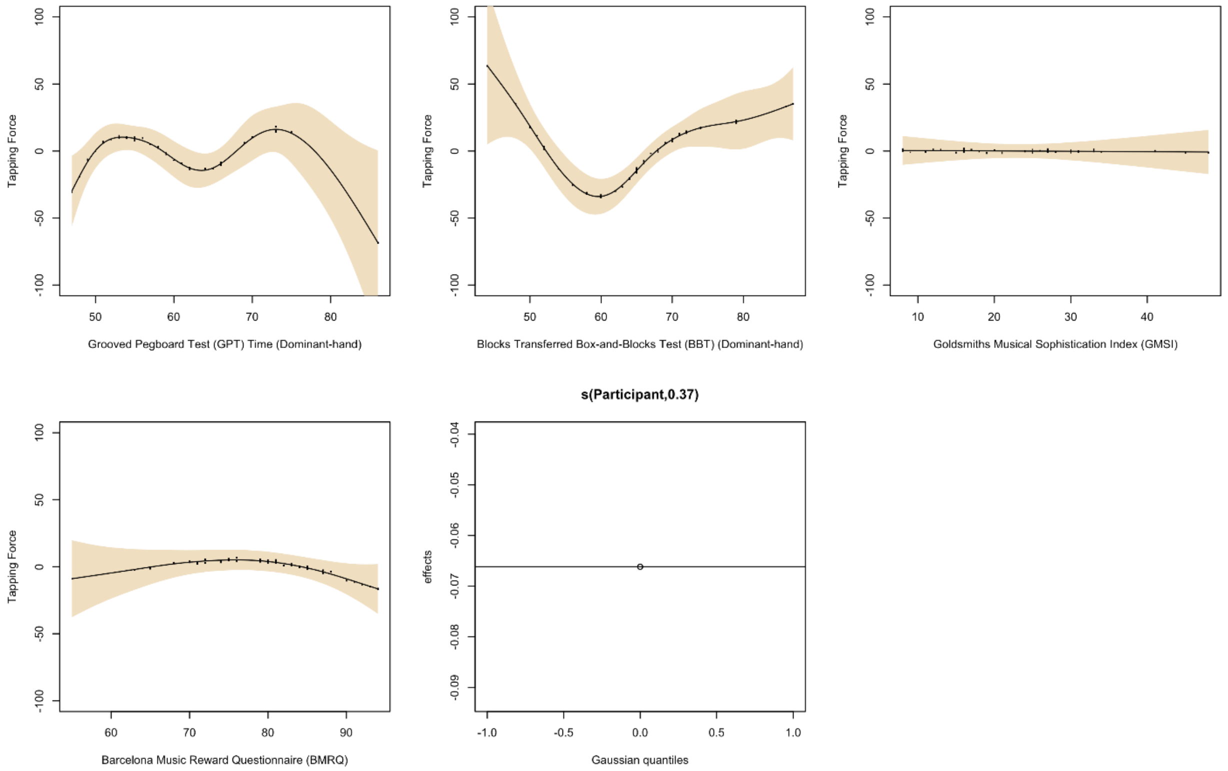

Note. Graphs visualize results when residual outliers are included. The solid line represents the fitted relationship, and the shaded area represents the 95% confidence interval of the estimated smooth effect.

Table X. *Results Summary GAM Motor and Musical Predictors and Tapping Consistency in the Dual Task.*

Smoothing terms		*Edf*	*df*	χ^2	*p*	*Bonferroni α*
s(GPT)		2.13	2.68	5.59	.166	.332
s(BBT)		1.00	1.00	3.09	.079	.158
s(GMSI MT)		1.00	1.00	11.55	<.001***	< .001***
s(BMRQ)		1.00	1.00	1.83	.176	.352
s(PPT)		$3.47x10^{-05}$	1.00	0.00	.344	.688
Parametric coefficients	Estimate	*SE*	*z*		*p*	*Bonferroni α*
(Intercept)	0.06	0.01	10.28		<.001***	< .001***
Auditory Cue	$-1.83x10^{-03}$	$3.56x10^{-03}$	-0.52		.607	1
R^2 (adj.)	-.062	Deviance explained			9.99%	

Note. Signif. codes: '***' 0.001 '**' 0.01 '*' 0.05. GPT = Grooved Pegboard Task calculated as time to complete in seconds; BBT= Box and Blocks Test calculated as total count of transferred blocks; GMSI MT= Musical Training subscale of the Gold Music Sophistication Index; BMRQ = total score on the Barcelona Music Rating Questionnaire; PPT = Participants.

Formula:
CV_DT ~ s(GPT_TIME_DH, k = -1) + s(BBT_DH_COUNT, k = -1) + s(GMSI_MT, k = -1) + s(BMRQ, k = -1) + Condition + s(PPT, bs = "re")

Figure F13. *Partial Effect Plots GAM Motor Predictors of Tapping Consistency in the Dual Task including Musical Questionnaires.*

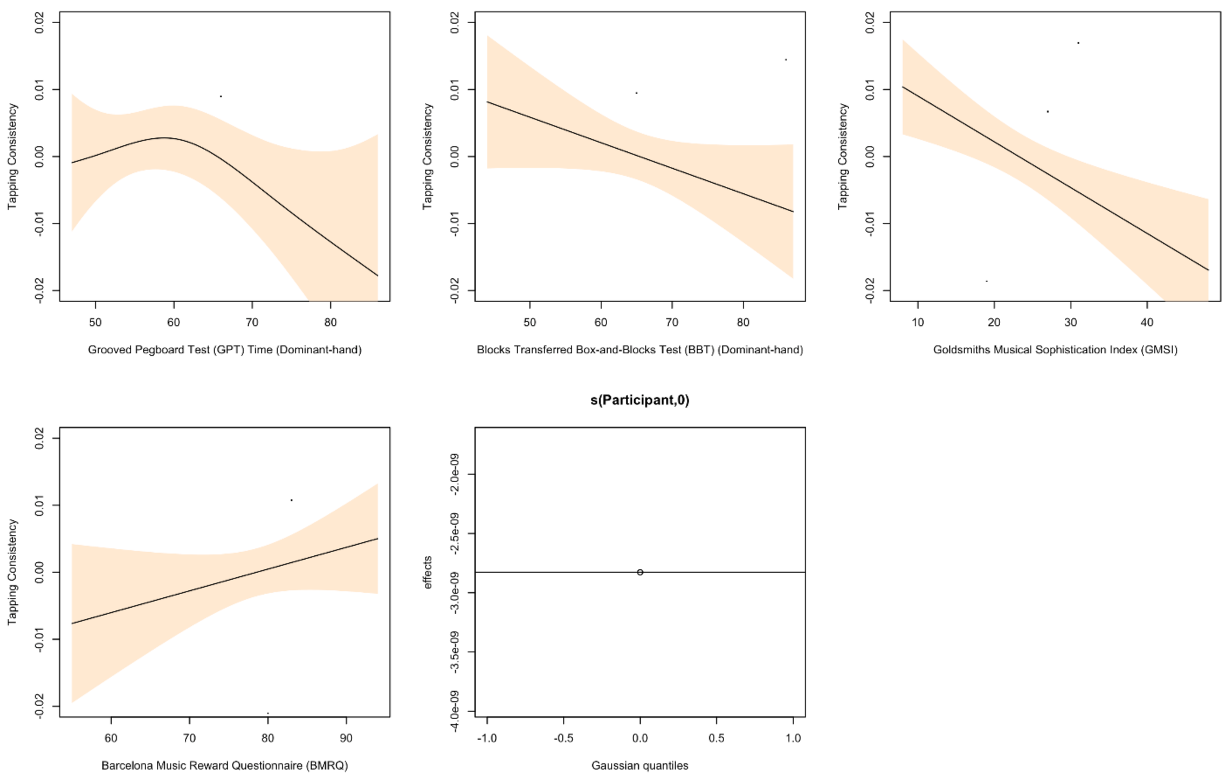

Note. Graphs visualize results when residual outliers are included. The solid line represents the fitted relationship, and the shaded area represents the 95% confidence interval of the estimated smooth effect.
